# Targeting EMT using low-dose Teniposide by downregulating ZEB2-driven activation of RNA polymerase I in breast cancer

**DOI:** 10.1038/s41419-024-06694-7

**Published:** 2024-05-08

**Authors:** Brandon J. Metge, Heba Allah M. Alsheikh, Sarah C. Kammerud, Dongquan Chen, Devika Das, N. Miranda Nebane, J. Robert Bostwick, Lalita A. Shevde, Rajeev S. Samant

**Affiliations:** 1 Department of Pathology, University of Alabama at Birmingham, Birmingham, AL USA; 2 Department of Medicine, University of Alabama at Birmingham, Birmingham, AL USA; 3 Birmingham VA Health Care System, Birmingham, AL USA; 4 High-Throughput Screening Center, Southern Research, Birmingham, AL USA; 5 O’Neal Comprehensive Cancer Center, University of Alabama at Birmingham, Birmingham, AL USA; 6 Present Address: Parexel Biotech, Waltham, MA USA

**Keywords:** Metastasis, Breast cancer, Target identification

## Abstract

Metastatic dissemination from the primary tumor is a complex process that requires crosstalk between tumor cells and the surrounding milieu and involves the interplay between numerous cellular-signaling programs. Epithelial–mesenchymal transition (EMT) remains at the forefront of orchestrating a shift in numerous cellular programs, such as stemness, drug resistance, and apoptosis that allow for successful metastasis. Till date, there is limited success in therapeutically targeting EMT. Utilizing a high throughput screen of FDA-approved compounds, we uncovered a novel role of the topoisomerase inhibitor, Teniposide, in reversing EMT. Here, we demonstrate Teniposide as a potent modulator of the EMT program, specifically through an IRF7–NMI mediated response. Furthermore, Teniposide significantly reduces the expression of the key EMT transcriptional regulator, Zinc Finger E-Box Binding Homeobox 2 (ZEB2). ZEB2 downregulation by Teniposide inhibited RNA polymerase I (Pol I) activity and rRNA biogenesis. Importantly, Teniposide treatment markedly reduced pulmonary colonization of breast cancer cells. We have uncovered a novel role of Teniposide, which when used at a very low concentration, mitigates mesenchymal-like invasive phenotype. Overall, its ability to target EMT and rRNA biogenesis makes Teniposide a viable candidate to be repurposed as a therapeutic option to restrict breast cancer metastases.

## Introduction

Metastasis is a multistep process wherein a cancer cell goes through dynamic restructuring of a broad range of cellular programs. Epithelial–mesenchymal transition (EMT) is a cellular plasticity program that plays a critical role in embryonic development and wound healing; however, several studies have demonstrated reactivation of the EMT program in cancer progression [1–3]. The precise contributions of EMT to invasion and metastasis have remained somewhat controversial, but its influence on stemness, drug resistance, apoptosis, senescence, and immunosuppression is very clear [1, 4–8]. Given the multifaceted effect of EMT on a plethora of cellular programs that drive cancer progression and drug resistance, it presents as an attractive target for therapeutic intervention. However, to date, the approach to targeting EMT remains elusive [9–11].

N-myc and STAT interactor (NMI) has been reported by us and others as a key regulator of pathways that promote tumor progression and metastasis [12–16]. Most notably, NMI loss promotes EMT through activation of TGF-β/SMAD as well as Wnt/β-catenin signaling in breast cancer and through transcriptional activation of p65 in gastric cancer [16–18]. As such, NMI serves as a pivotal regulatory node for EMT. Our assessments revealed that NMI expression is severely compromised in advanced-stage breast cancer. However, this compromised expression was not due to NMI gene deletion or mutations. Therefore, we hypothesized that restoration of NMI expression could serve as a viable treatment modality to limit the metastatic progression of breast cancer.

Here, we present findings from a high throughput screen of bioactive compounds, including FDA-approved drugs, that revealed novel inhibitors of EMT. We identified topoisomerase inhibitors as a major class of compounds that induce NMI expression. Specifically, Teniposide effectively induced NMI expression and caused a dramatic reduction in the mesenchymal-like attributes of breast cancer cells. We noted that this effect of Teniposide was predominantly downstream of increased NMI expression that prompts reduced expression of the key EMT transcription factor Zinc Finger E-Box Binding Homeobox 2 (ZEB2). Unexpectedly, we observed that the decrease in the mesenchymal-like attributes was concomitant with inhibition of ribosomal DNA (rDNA) transcription. We demonstrate that ZEB2 binds to rDNA promoter and regulates rDNA transcription. Teniposide reduced ZEB2 levels leading to downregulated rDNA transcription and mesenchymal attributes of breast cancer cells. We also demonstrate that at low concentrations, Teniposide effectively reduces lung colonization of breast cancer.

## Methods

### Cell culture

T47D cells were maintained in RPMI-1640 (Thermo Fisher, Waltham, MA, USA) media supplemented with 10% FBS (Thermo Fisher) and 10 µg/mL insulin (Sigma-Aldrich, St. Louis, MO, USA). MDA-MB-231 cells were cultured in DMEM/F-12 supplemented with 5% FBS. MDA-MB-231-1833 (a clonal line derived as a bone metastatic variant of MDA-MB-231: *referred here as 1833*) was a kind gift from Yibin Kang [19] and was maintained in DMEM/F-12 supplemented with 10% FBS.

### Generation of stable cell lines

To generate NMI reporter-MDA-MB-231 cell line (NMI-619), MDA-MB-231 cells were transfected with pGL4-NMI-619 plasmid and selected in 500 ng/mL puromycin (Millipore Sigma, Burlington, MA, USA). T47D cells silenced for NMI were generated as previously described [17].

1833 cells expressing luciferase and GFP were generated using lentiviral particles containing an expression vector with firefly luciferase and GFP (Genecopeia, Rockville, MD). Stable cell lines were selected using puromycin at 500 ng/mL. 1833 cells stably expressing HA-tagged NMI were generated by transfection of 1833 with pIRESpuro3-HA-NMI. The resultant cells were maintained in media containing 500 ng/mL puromycin. 1833 cells expressing inducible shRNA targeting NMI were generated using lentivirus with pTRIPZ shNMI co-expressing RFP (Horizon Discovery, Waterbeach, UK). Stable cell lines were generated by sorting for RFP positivity following induction with 2 μg/mL doxycycline (Millipore Sigma). The resulting cells were maintained in 500 ng/mL puromycin (Millipore Sigma).

### Cloning

For generating the NMI reporter construct, a 619 base pair region (Supplementary Information: Sequence #1) of the NMI promoter was inserted into the KpnI and NheI sites of the pGL4 luciferase reporter plasmid (Promega, Madison, WI).

### High throughput screening

A collection of 5195 bioactive compound samples was compiled at Southern Research (High-Throughput Screening Center, Southern Research, AL, USA) from various vendors (Enzo, Microsource, Selleck, Prestwick). The collection included 2137 FDA-approved drugs, many of which were repeated as different samples coming from different vendors. Compounds and DMSO carrier were dispensed into 384-well white opaque-bottom plates (3570BC, Corning, Corning, NY, USA) in 5 µL volume using the Biomek FX liquid handler (Beckman Coulter). In total, 25 µL of NMI-619 luciferase reporter cells were added to the plates for a final count of 10,000 cells/well, a final compound concentration of 20 µg/mL, and a final DMSO concentration of 0.4%. Cells treated with 0.4% DMSO served as the negative control and cells treated with 200 ng/mL IFNγ (R&D Systems, Minneapolis, MN) served as the positive control. Plates were incubated at 37 °C in 5% CO_2_ for 24 h in a humidified atmosphere and luminescence was analyzed with Bright-Glo (Promega) on an Envision plate reader (PerkinElmer, Waltham, MA, USA). Percent activation was calculated as 100 × ((Compound luminescence value − Median Negative control)/(Median Positive (IFNγ) control − Median Negative control)). A total of 5195 compounds were tested on 2 separate days, with a Pearson’s correlation of 0.96 between the two runs. The Average Z′ factor for all 36 plates screened (18 plates/run) was 0.8, with Z′ > 0.7 for all plates.

### Luciferase assay

NMI-619 reporter cells were seeded at 10,000 cells/well into a 96-well plate and allowed to attach overnight. The next day, the media was changed to complete media with the compounds as indicated and incubated at 37 °C for 24 h. After incubation, cells were washed with PBS and lysed in 30 µL reporter lysis buffer. Cell lysates underwent a single freeze-thaw cycle. Luminescence was measured with the firefly assay reagent (Promega) using a Promega 20/20 luminometer. Luciferase was normalized against total protein in each well, and each compound was assayed in triplicate.

### Gene expression analysis by RNA-seq

RNA was isolated from mammary tumors from MMTV-Neu Nmi wild-type (*n* = 3) and MMTV-Neu Nmi-knockout (NmiKO) mice (*n* = 4), using the RNeasy Mini Kit as per the manufacturer’s protocol. Next-gen sequencing was performed by GENEWIZ Inc. (Chelmsford, MA). Briefly, the RNA was quantified using Qubit 2.0 Fluorometer (ThermoFisher Scientific, Waltham, MA, USA). The integrity of RNA was assessed using TapeStation (Agilent Technologies, Palo Alto, CA, USA).

NEBNext Ultra II RNA Library Prep Kit for Illumina (New England Biolabs, Ipswich, MA, USA) was used per the manufacturer’s instructions for the preparation of RNA sequencing library. Enrichment of mRNA was done using Oligod(T) beads. Following a 15-min fragmentation (94 °C), the enriched mRNAs were used for first-strand and second-strand cDNA synthesis. Universal adapters were ligated to cDNA fragments following end repair and 3′end adenylation. Following this, index addition and library enrichment were performed using PCR (limited cycles). The library was validated on the Agilent TapeStation (Agilent Technologies, Palo Alto, CA, USA), and quantified using Qubit 2.0 Fluorometer (ThermoFisher Scientific, Waltham, MA, USA) and also by Q-PCR (KAPA Biosystems, Wilmington, MA, USA). The sequencing libraries were multiplexed and clustered onto a flow cell. The samples were sequenced using Illumina HiSeq 4000 (per the manufacturer’s instructions), using a 2 × 150 bp paired-end configuration. The HiSeq Control Software (HCS) was used for image analysis and base calling. Raw sequence data (.bcl files) generated from Illumina HiSeq was converted into fastq files and de-multiplexed using Illumina bcl2fastq 2.20 software. One mis-match was permitted for index sequence identification. Trimmomatic tool [20] was used for removing adapter sequences from raw data in fastq format. Alignment was done using HISAT2 [21] algorithm, and PCR replicates were removed using SAMtools [22] before expression level quantification. The resulting BAM or SAM files were analyzed through a workflow of Partek Genomics Suite (PGS, St Louis, MO, USA). Reads per kilobase of transcript per million mapped reads normalization [23] was performed prior to statistical analysis to select genes by fold changes and statistical *p* values. Additional pathway analysis was conducted by using Ingenuity Pathway Analysis (IPA, Redwood City, CA USA).

For gene set enrichment analysis (GSEA), expression data were log base 2 normalized using Morpheus (https://software.broadinstitute.org/morpheus/). Subsequently, GSEA was performed utilizing GSEA v4.2.3 Mac App using a collection of MSigDB annotated gene sets [24]. Annotations from GSEA were illustrated as bubble plots using the “ggplot2” package in RStudio v4.1.2 (Boston, MA, USA).

The sequence data are deposited in Sequence Read Archive database: BioProject accession number-PRJNA853372.

### 3D assay for growth morphology

Cells in growth media supplemented with 2% Cultrex 3D culture matrix (R&D systems, Minneapolis, MN) were seeded onto 8-chambered glass slides (Millipore Sigma) coated with 150 µL Cultrex and incubated at 37 °C in 5% CO_2_. Media was replaced every 2 days with fresh complete media containing 2% Culturex 3D culture matrix. On the 5th day after seeding, cells were treated with Teniposide (500 nM or 1 µM). For doxycycline treatment, cells were pretreated in 2 mg/mL doxycycline 2 days prior to seeding in 3D assay and then maintained in 3D culture during the experiment. Images were taken between days 7 and 10 after seeding, using a Nikon eclipse Ti microscope. Laminin-V was visualized using Alexa Fluor 488-conjugated anti-laminin-V (clone D4B5) (Millipore Sigma) and nuclei were stained with Vectashield Mounting Medium containing DAPI (Vector Laboratories, Inc., Burlingame, CA, USA). Image J analysis software was used to measure circularity and area of spheres.

### Migration assay

Polyethylene terephthalate track-etched cell culture insert filters with 8-micron pores (Corning, Corning, NY) were coated with 6 ng/ml gelatin (Sigma, St. Louis, MO) overnight. The following morning, the filters were rehydrated in serum-free media. MDA-MB-231 cells (50,000) that had been pretreated for 24 h with vehicle control or 500 nM Teniposide were seeded in each insert, and the inserts were placed in wells with serum-containing media with vehicle control or Teniposide. After 4 h at 37 °C, the media was removed from the inserts and the inserts were placed in 4% paraformaldehyde. Cells were stained with 0.5% crystal violet in 25% methanol, then rinsed in ultrapure water to remove excess stain. Images were captured using a Nikon Eclipse Ti-U, and cells were quantified using ImageJ.

### Invasion assay

Corning Biocoat Matrigel invasion chambers were thawed and rehydrated with serum-free media. MDA-MB-231 cells were pretreated for 24 h with vehicle control or 500 nM Teniposide and then 15,000 cells were seeded in each chamber in serum-free media. Chambers were then placed in wells containing media with 10 µg/ml fibronectin. After 16 h, the media was removed from the inserts and the inserts placed in 4% paraformaldehyde. Cells were stained with 0.5% crystal violet in 25% methanol, then rinsed in ultrapure water to remove excess stain. Images were captured using a Nikon Eclipse Ti-U, and cells were quantified using ImageJ.

### Wound healing assay

MDA-MB-231 cells (150,000) were seeded in 6-well plates and treated with vehicle control or 500 nM Teniposide. After 24 h of treatment, a 200 µl pipet tip was used to create a scratch down the center of each well. Wells were washed gently, then treatment-containing media was added back to each well. Images were captured using a Nikon Eclipse Ti-U at 0, 4, 8, and 24 h. Distance migrated was measured using Nikon Elements software and graphed using Graph Pad Prism (GraphPad Software, San Diego, CA).

### Cell viability assays

#### CyQuant assay

MDA-MB-231 cells were seeded at 10,000 cells/well in a 96-well plate in triplicate and incubated at 37 °C. Cells were subsequently treated with 500 nM, 1 µM, 50 µM Teniposide or vehicle control for 24 h. Cell viability was determined by CyQUANT Direct Cell Proliferation Assay (Thermo Fisher) as per manufacturer’s protocol measuring fluorescence at excitation (EX) 485/20 nm and emission (EM) 528/25 nm using BioTek Flx800 (Aligent Technologies Santa Clara, CA).

#### Flow cytometry

MDA-MB-231 cells were treated with 500 nM, 1 µM, 50 µM Teniposide or vehicle control and cells were assessed for viability using Zombie Green Fixable Viability Kit (Biolegend, San Diego, CA). Briefly, cells were harvested at various time points and washed in 1× PBS with 2% FBS and then incubated at room temperature for 15 min in Zombie Green Fixable Viability dye diluted 1:1000. Cells were then washed in 1× PBS and resuspended in FACS buffer. The cells were analyzed using BD Accuri C6 Plus (BD, Franklin Lakes, NJ) and analysis was completed using FlowJo (FlowJo, Ashland, OR).

### Determining combination index (Ci)

MDA-MB-231 cells were plated (10,000 cells/well) in a 96-well plate and allowed to attach overnight. Cells were then treated with 0.5 µM Teniposide for 24 h. Following this, cells were treated with a range of concentrations of Doxorubicin alone or as a combination of Doxorubicin and 0.5 µM Teniposide. After 48 h the assay was terminated using Cell Titer Fluor (Promega) as per the manufacturer’s instructions. The combination index was determined using CompuSyn software V 1.0 (ComboSyn Inc., Paramus, NJ, USA) Ci < 1 is considered synergistic, whereas antagonistic drug action has Ci > 1, or no effect is Ci = 0.

### Chromatin immunoprecipitation assay (ChIP)

Cells treated with Teniposide were processed using the SimpleChIP Plus Enzymatic kit (Cell Signaling, Danvers, MA) as per manufacturer’s protocol. Briefly, cells were fixed with 1% formaldehyde at room temperature. Then nuclei were isolated followed by chromatin digestion using micrococcal nuclease and sonication. Cross-linked chromatin (10 μg) was immunoprecipitated using 2 μg of anti-IRF-7 (Cell Signaling) or anti-ZEB2 (Bethyl Labs, Montgomery, TX). The precipitated chromatin was then eluted from the precipitate and cross-links were reversed. The immunoprecipitated DNA and input were analyzed by RT-qPCR using 2× Maxima SYBR Green Master Mix (Thermo Fisher). To detect interferon regulatory factor 7 (IRF7) occupancy at the NMI basal promoter elements, the following primer pairs were used:

For-CGGAAACTTCAGGTATACTTC, Rev-CTGCTTTAACGGCGATTTTTC.

To detect ZEB2 occupancy at the rDNA, the primer pairs used were as follows:

For-GTACTTTTAGTAGAGACGGTG, Rev-CACTTTGGGAGGCTAAGGC.

Threshold cycle (C[T]) values of input DNA were used to calculate the percent input of immunoprecipitation. Percent Input = 2% × 2^(C[T] 2% Input Sample−C[T] IP Sample)^. Percent enrichment in comparison with corresponding controls is depicted. Each reaction was done in triplicate using an Applied Biosystems StepOnePlus (Thermo Fisher).

### Real-time PCR

RNA was isolated from cells using the RNeasy Mini Kit (Qiagen, Hilden, Germany). One microgram total RNA was used to generate cDNA using High-Capacity cDNA kit (Thermo Fisher). Real-time PCR was performed using 40 ng total cDNA in each reaction, along with 2× TaqMan Fast Advance Master Mix (Thermo Fisher) and the following TaqMan primer probes: β-actin, NMI, CDH1, CDH2, KRT14, SNAI1, SNAI2, VIM, ZEB1, ZEB2 (Thermo Fisher).

RNA polymerase I (Pol I) transcription activity was quantitated by monitoring levels of 5′ external transcribed spacer (ETS) of the 47S pre-RNA using RT-PCR. cDNA (1:50 dilution) was used with 2× Maxima SYBR Green Master Mix (Thermo Fisher) along with the following primer sets to perform the reaction [25]:

Human 5′ETS 851–961 For-GAACGGTGGTGTGTCGTT, Rev-GCGTCTCGTCTCGTCTCACT; β-Actin For-CATGTACGTTGCTATCCAGGC, Rev-CTCCTTAATGTCACGCACGAT.

Mouse 45S rRNA ITS1 For-CCGGCTTGCCCGATTT, Rev-GGCCAGCAGGAACGA; β-Actin For-GGCTGTATTCCCCTCCATCG, Rev-CCAGTTGGTAACAATGCCATGT.

Applied Biosystems StepOnePlus Real-time PCR machine was used for the reactions (done in triplicate). Analysis was done using ^ΔΔ^CT to determine relative fold changes in mRNA or 5′ETS transcripts.

### Real-time PCR arrays

Targeted pathway analysis for EMT-relevant gene signatures was carried out using Qiagen Human EMT RT^2^ Profiler PCR Arrays (Qiagen). cDNA was generated using 500 ng total RNA and RT^2^ First Strand Kit (Qiagen). An equal amount of cDNA was added to a 96-well plate containing SYBR Green-optimized primer assays. Each assay was conducted in duplicate and analysis was done using Gene Globe RT^2^ Profiler PCR Data Analysis website (Qiagen).

### EU incorporation assay

Incorporation of 5-ethynyl uridine (EU) into nascent rRNA transcripts following a short EU pulse was measured as a readout of rRNA synthesis, as described previously [26]. Cells were seeded (overnight) onto glass coverslips. Where indicated, Teniposide treatment was done for 24 h, after which cells were pulsed with 1 mM EU (Thermo Fisher) for 3 h. Cells were then fixed with 3.7% formaldehyde and EU-incorporated RNA transcripts were labeled using Click-iT RNA Alexa Fluor 488 HCS Assay (Thermo Fisher) as per manufacturer’s protocol. Briefly, cells were washed and permeabilized with 0.5% Triton X-100 for 15 min and then incubated in Click-iT reaction cocktail 30 min at room temperature. Co-staining with fibrillarin was done immediately following Click-iT labeling. Cells were blocked in 5% BSA for 1 h at room temperature, followed by incubation at 4 °C overnight, with anti-Fibrillarin antibody (Abcam, Cambridge, UK). Alexa Fluor 594 secondary antibody (Thermo Fisher) was used for detection and the specimen was mounted using Vectashield Plus with DAPI (Vector Labs, Burlingame, CA, USA). Images were acquired using a Nikon Eclipse Ti-U microscope (Nikon Instruments Inc., Melville, NY, USA). Exposure times for all images were identical. Mean fluorescence intensity of 488 and 594 binary intersection was determined using NIS Elements Advanced Research software. Eight random fields were analyzed. Representative images are depicted.

### Immunohistochemistry

Immunohistochemical detection was performed using the Dako Envision + Dual Link System-HRP (Agilent, Santa Clara, CA) according to manufacturer’s protocol. Briefly, human breast cancer tissue microarray (BRM961a and BRM961b from US Biomax, Derwood, MD) were probed with anti-NMI 1:3000 antibody (Millipore Sigma) overnight at 4 °C, followed by secondary antibody for 1 h at RT, then mounted in Cytoseal (Thermo Scientific, Waltham, MA) mounting medium. Images were captured (40×) using a Nikon Eclipse Ti inverted microscope. Staining was assessed using light microscopy and quantitated using immunoreactive scoring [27].

### Immunofluorescence

MDA-MB-231 cells plated on a coverslip were fixed in 4% paraformaldehyde, followed by a rinse with 1× PBS prior to permeabilization in 1× PBS with 0.3% triton X-100. Cells were incubated in blocking buffer (1× PBS with 0.3% triton X-100 and 5% BSA) and then incubated overnight in primary antibodies (anti-ZEB2, Sigma-Aldrich and anti-Fibrillarin, Abcam) diluted in 1× PBS with 0.3% triton X-100 and 1% BSA at 4 °C. The next day, cells were washed in 1× PBS, then incubated in secondary antibodies (Alexa Fluor 594 goat anti-rabbit and Alexa Fluor 488 goat anti-mouse, Thermo Fisher) diluted in blocking buffer. After washing in 1× PBS, the coverslip was mounted using VECTASHIELD PLUS Antifade Mounting Medium with DAPI (Vector Laboratories). Nikon Eclipse Ti-U microscope was used to visualize the cells.

### Nucleolar fractionation

MDA-MB-231 cells were treated with 1 µM Teniposide or control (DMSO) for 24 h. Nucleoli were isolated from the cells as previously described [28]. Briefly, the cells were washed and collected in a minimal volume of 1× PBS. They were subjected to osmotic shock prior to lysis in NP-40-containing buffer. Cells were then subjected to homogenization using a Dounce homogenizer. Nuclei were purified through a 250 mM sucrose cushion and then sonicated to release the nucleoli. Nucleoli were purified through a 340 mM sucrose cushion, then resuspended in 1× RIPA buffer with HALT^TM^ protease and phosphatase inhibitor.

### Immunoprecipitation and western blotting

Cells were lysed using ice-cold RIPA lysis buffer (Millipore Sigma) with HALT protease and phosphatase inhibitors (Thermo Fisher). For western blot analysis, cell lysates were resolved using SDS-PAGE and transferred to PVDF membrane. Membrane was blocked in 5% non-fat dry milk in Tris-buffered saline with 0.1% Tween-20 (TBST) and incubated with primary antibody overnight at 4 °C. Membrane was then washed in TBST and incubated with specific HRP-conjugated secondary antibody. The signal was visualized using ECL Prime (GE Healthcare, Pittsburgh, PA) and detected using the GE Amersham 600 Imager (GE Healthcare). The following antibodies were used: anti-α-tubulin-HRP (Cell Signaling), anti-NMI (Millipore Sigma), γH2AX (Cell Signaling), anti-IRF-7 (Cell Signaling), anti-ZEB2 (Bethyl Labs), anti-ZEB1 (Novus Biologicals, Littleton, CO), and anti-Fibrillarin (Abcam).

### Pulmonary metastasis assays (PuMA)

PuMA was carried out using a procedure adopted from a previous report by Mendoza et al. [29]. Two hundred thousand untreated or pretreated (500 nM Teniposide for 48 h) GFP-expressing 1833 cells were injected into the lateral tail vein of nude mice. Fifteen minutes post injection, mice were euthanized, and lungs were cannulated and perfused with 0.6% agarose M-199 media supplemented with 1.0 μg/mL crystalline bovine insulin, 0.1 μg/mL hydrocortisone, 0.1 μg/mL retinyl acetate, 100 U/mL penicillin and 100 μg/mL streptomycin, and 7.5% sodium bicarbonate. Lungs with solidified agarose were thinly sliced and placed on a 2 × 2 × 0.7 cm piece of Surgifoam (Ethicon, Somerville, NJ). The Surgifoam pieces were pre-soaked in culture media. Media was replaced every other day (±500 nM Teniposide), and sections were flipped over with each media change. For those assays done with pretreated cells, lung sections were maintained in media devoid of Teniposide. Pictures were captured using an SMZ800 stereo zoom microscope (Nikon), and mean fluorescent area was determined using NIS Elements software (Nikon).

Pharmacokinetics and pharmacodynamics for Teniposide (VM26) are well documented (per-“Accessdata”-data for FDA-approved drugs) https://www.accessdata.fda.gov/drugsatfda_docs/label/2011/020119s010s011lbl.pdf. In patients, the approved doses show side effects. In mice, (Lewis lung carcinoma) a dose of 6.5 mg/kg × 3 times/week was found to be very effective at tumor reduction, with few limited side effects/toxicity [24, 30]. Per our knowledge, the Teniposide dose used in this study (500 nM) is much below any concentrations used in previous studies.

### Ethical approval of the animal study protocols

The animal facility at the University of Alabama at Birmingham (UAB) is AALAC accredited. All animal use mentioned in this study was approved by Institutional Animal Care and Use Committees (IACUC). All mice were maintained in the UAB animal facility in accordance with the guidelines of the IACUC. The animal use was permitted under IACUC protocol numbers: IACUC-21248 for PuMA, IACUC-10095 for NmiKO mice generation and breeding, and IACUC-10011 for NmiKO mice tumor studies on MMTV-Neu background.

### Collection of mammary tumor tissue and RNA from Nmi mammary tissue-specific knockout mice

Tumor-bearing, mammary tissue-specific NmiKO mice on MMTV-Neu background are previously described [16]. RNA was extracted using Qiagen RNeasy Mini Kit as per manufacturer’s protocol.

### Informatics analysis using public data sets

Public data portal (https://xenabrowser.net) was used to access RNA sequencing data (IlluminaHiSeq) from 1247 primary breast cancer tumors (TCGA Breast Cancer). Data for NMI, ZEB1, and ZEB2 and 10-Year KM overall survival were extracted and stratified as high or low NMI, ZEB1, or ZEB2 based on division of data into quartiles, with top 25% as high and bottom 25% as low expression. Additionally, patients were further stratified into high NMI/low ZEB2 or low NMI/high ZEB2. Survival curves were generated using Graph Pad Prism software. Comparisons were considered statistically significant for *p* value < 0.05.

Data from GSE37975 were downloaded from NCBI Gene Expression Omnibus (https://www.ncbi.nlm.nih.gov/geo) and analyzed for fold changes of Nmi and Irf7 transcript. Similarly, data from GSE37828 were analyzed for Nmi and Irf7 transcript abundance.

### Statistics

All data presented are a representation of experimental repeats. All statistical tests are indicated in figure legends with associated “*p* values”. All statistical analysis was carried out using GraphPad Prism version 9. Additional statistical analyses are embedded in alignment, quantification, and comparison in GSEA (broadinstitute.org) analysis and gene expression analysis by Partek (Illumina.com).

## Results

### Compromised expression of NMI drives breast cancer progression

**Fig. 1:**
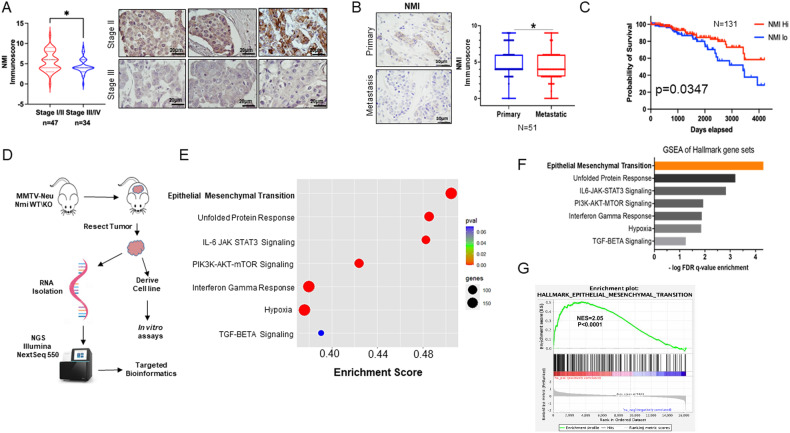
Compromised expression of NMI drives breast cancer progression. **A** Immunoscore of NMI protein staining in stage I/II (*n* = 47) versus stage III/IV (*n* = 34) from breast cancer tissue array is represented as a violin plot. Adjacent panel shows representative images of specimens from stages II and III stained for NMI. “*” represents that the comparison is statistically significant (*p* < 0.05). **B** Immunoscore of NMI protein staining in matched primary tumor (*n* = 51) versus LN metastases (*n* = 51) from breast cancer tissue array. “*” represents that comparison is statistically significant (*p* = 0.02). **C** TCGA breast cancer patient data representing survival probability in patients with high NMI expression versus low NMI expression *n* = 131 and *p* = 0.04, log-rank test statistics = 4.333. **D** Workflow scheme for RNA-seq of Nmi fl/fl MMTV-Neu (WT) and Nmi fl/fl Cre^+(KRT14)^ MMTV-Neu (NMIKO) tumors and tumor-derived cells. **E** Bubble plot of GSEA using MSigDB Collection Hallmark Gene Set. Diameter of the circles depicts relative number of genes in each associated gene set, and red indicates most significant enrichment score. EMT gene set is the most significantly enriched gene set. **F** Bar graph of GSEA Hallmark gene sets, depicts EMT as the topmost significantly enriched gene set in Nmi-knockout tumors. **G** The plot shows the enrichment and quantitative details of the EMT signature from GSEA Hallmark gene sets, NES = 2.05, *p* < 0.0001.

Multiple studies from us as well as others have underscored that NMI functionally impedes mesenchymal transition and reduces metastasis [12, 15–18]. To further resolve the clinically significant role of NMI in the context of tumor progression, we undertook a detailed analysis of breast tumors in the perspective of stages of disease progression – stages I–IV. Immunohistochemical staining of NMI across these stages revealed a noticeable reduction in the abundance of NMI protein with advanced stage. Specifically, breast tumors from stages III and IV patients showed starkly reduced expression of NMI compared to early stages (stages I and II) (Fig. 1A). This observation implied that loss of NMI expression may be associated with metastasis. Therefore, we evaluated NMI immunohistochemically from primary breast tumors and their corresponding metastasis and found that NMI protein expression was significantly decreased in metastatic tissues (Fig. 1B). Overall, our data highlight that metastatic progression is associated with compromised expression of NMI.

TCGA data from breast cancer cohorts did not show abundance of any specific deletion or mutation events in the NMI gene. Given the low abundance of NMI in metastatic tissues, we sought to determine the correlation between NMI expression and survival. NMI expression data were acquired from gene expression array AgilentG4502A_07_3 TCGA Hub (Xenabrowser) [31]. NMI expression and 10-year Kaplan–Meier overall survival were extracted for analysis. Patients were stratified by quartile into top and bottom for NMI expression (*N* = 131). Patients with abundant NMI transcript level showed much better prognosis in terms of higher survival probability in contrast with patients with lower abundance of NMI transcript, who clearly showed worse survival probability (Fig. 1C).

To understand the consequences of compromised expression of NMI in relation to tumor progression, we recapitulated its loss in a mammary-specific NmiKO mouse model. This model clearly demonstrated that in mammary tumors that lacked Nmi expression, there is an increased incidence of metastasis [16]. To obtain mechanistic insight, we analyzed RNA from NmiKO mammary tumors by RNA-seq (Fig. 1D). GSEA utilizing Hallmarks MSigDB collections gene sets revealed highly significant enrichment of gene sets across a number of important signaling pathways related to cancer hallmarks. Given NMI’s pivotal role in STAT signaling or interferon response, it was important to note that both these signaling pathways presented as significantly enriched gene sets in NmiKO mice. Strikingly, the most significantly enriched gene set in NmiKO mice corresponded to EMT (Fig. 1E, F, and G). Consistent with earlier reports in the context of human breast cancer cells [32], this observation independently endorsed that Nmi loss has a robust effect on the aberrant activation of the EMT program. Taken together, these results highlight the potential impact of absence of NMI expression on metastatic progression.

### NMI expression is increased by Teniposide

**Fig. 2:**
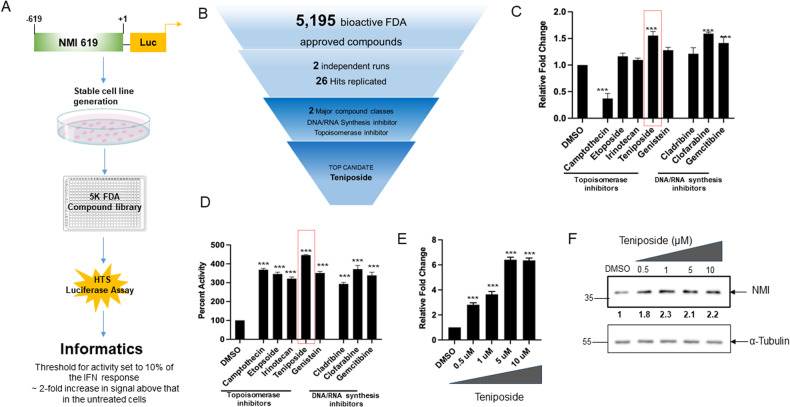
NMI expression is increased by Teniposide. **A** Schematic of high throughput screen (HTS) workflow using NMI promoter. **B** Schematic representing approach to narrowing down to Teniposide as the top candidate from the HTS. **C** Real-time PCR of NMI expression in MDA-MB-231 treated with 1 µM of named compounds. The experiment was conducted in triplicates and repeated one time. **D** Luciferase activity assay of MDA-MB-231 NMI-619 cells treated with two major class compounds from the HTS. Percent activity was calculated in relation to DMSO control. The experiment was conducted in triplicates and repeated one time. **E** NMI mRNA expression in MDA-MB-231 cells treated with increasing concentrations of Teniposide. The experiment was conducted in triplicates and repeated one time. **F** Western blot analysis of NMI protein levels in MDA-MB-231 cells treated with increasing concentrations of Teniposide. Numerical values (in bold) represent quantitation estimate of increased NMI protein expression normalized to α-tubulin loading control in treatment groups versus control. Data are presented as mean + SEM. Significance determined by Dunnett’s multiple comparison tests. ***p < 0.001.

We hypothesized that small molecule compounds that can increase the expression of NMI may antagonize mesenchymal-like attributes of breast cancer cells. Thus, we undertook a focused screen of FDA-approved compounds to identify small molecules that can upregulate the expression of NMI (summarized in Fig. 2A). Previous studies have demonstrated that NMI expression is compromised in breast cancer cell lines with mesenchymal-like attributes [15, 17]. A classic mesenchymal-like breast cancer cell line MDA-MB-231 has practically undetectable levels of NMI protein compared to the epithelial-like breast cancer cell line, T47D (Supplementary Fig. 1A). Our previous work has established that in MDA-MB-231 cells protein levels of NMI are dramatically elevated following treatment with interferon-γ (IFNγ) [15]. Thus, we decided to use MDA-MB-231 cells as a screening tool for small molecule compounds that can increase the expression of NMI. To accomplish our screening goal, we cloned a 619 base pair fragment of the human NMI proximal promoter into a luciferase reporter vector and subsequently generated a stable reporter cell line (NMI-619). Informed by the fact that IFNγ activates NMI transcript expression [15, 33], we used IFNγ to assess reproducibility of our screening assay (Supplementary Fig. 1B). A library consisting of 5195 FDA-approved bioactive compounds was screened using twofold induction of NMI promoter activation as a cut-off for positive hits. This screen was conducted and then independently repeated for rigor and consistency of observations. Of the 5195 compounds, 26 hits were identified which consisted of two major classes of compounds: DNA/RNA synthesis inhibitors and topoisomerase inhibitors (Fig. 2B). These two classes consisted of eight unique compounds (Supplementary Fig. 1C).

Further evaluation of target compounds by real-time quantitative PCR (Fig. 2C) and luciferase assay (Fig. 2D) revealed Teniposide as the most proficient transcriptional activator of NMI. We then independently tested the effect of a range of concentrations of Teniposide and observed that even at low micromolar concentration (0.5–1 µM) Teniposide was able to elicit robust transcriptional activation as evidenced by an approximately fourfold increase in NMI transcript levels (Fig. 2E) and approximately twofold increase in NMI protein expression (Fig. 2F).

Topoisomerase inhibitors are known to induce DNA breaks and thereby elicit downstream signaling pathways in response to DNA damage, along with adverse effects on cell viability. Cells treated with low concentrations (0.5–1 µM) of Teniposide showed very weak γH2AX induction (Supplementary Fig. 1D). Cell viability assessment using two independent methods, flow cytometry based Zombie green^TM^ (Supplementary Fig. 1E) and CyQUANT^TM^ Direct Cell Proliferation Assay (Supplementary Fig. 1F) demonstrated that low concentrations (0.5–1 µM) of Teniposide minimally impact cell viability.

### Teniposide upregulates NMI via IRF7

**Fig. 3:**
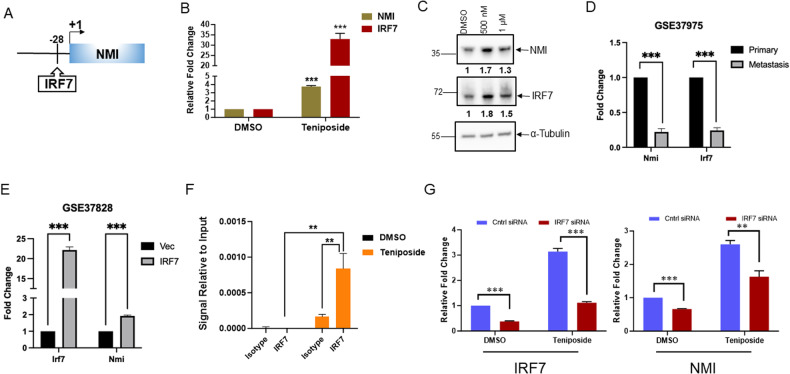
Teniposide upregulates NMI via IRF7. **A** Schematic of NMI promoter representing putative IRF7 recognition site at −28 position. **B** Real-time PCR estimation of NMI and IRF7 levels following 500 nM Teniposide treatment. IRF7 and NMI both respond to the treatment. *p* = 0.0001. **C** Western blot analysis of NMI and IRF7 protein expression after 250 or 500 nM Teniposide treatment. Values represent densitometry of NMI or IRF7 signal intensity normalized to respective α-tubulin loading control in treatment groups versus control. **D** Expression levels of Nmi and Irf7 in 4T1 primary tumor versus bone metastasis from Geo dataset GSE37975. **E** Expression level of Nmi in Irf7-overexpressing cells demonstrating Irf7-induced Nmi expression from Geo dataset GSE37828. **F** ChIP of putative IRF7 site in NMI promoter sequence in MDA-MB-231 cells treated with Teniposide (500 nM). Signal relative to 2% input for isotype and IRF7. **G** Real-time PCR for IRF7 and NMI levels in MDA-MB-231 cells silenced for IRF7 following Teniposide treatment (500 nM). Data are presented as mean + SEM. *p* determined by Student’s *t* tests. **p < 0.01, ***p < 0.001.

Studies have identified that NMI expression is transcriptionally induced by IFNγ [12]. IFNγ activates a plethora of cellular responses. IRF7 is a key transcription activator regulated by IFNγ [34, 35]. Our scan of the NMI promoter sequence revealed a putative IRF7 binding site at position −28 (Fig. 3A). Thus, we hypothesized that IRF7 could be a transcription activator of NMI. In breast cancer cells treated with low-concentration Teniposide, both IRF7 and NMI were significantly upregulated at the transcript (Fig. 3B) and protein level (Fig. 3C). Seminal work by Bidwell et al. confirmed the importance of IRF7 in mediating bone metastasis in a murine mammary tumor model [36]. We examined RNA-seq data (GSE37975) from that publication and identified that NMI and IRF7 expressions display concurrent trends in primary tumors and in metastases (Fig. 3D). Furthermore, query of RNA-Seq data by Bidwell et al. following ectopic expression of IRF7 (GSE37828) shows elevated NMI expression in the IRF7 expressor group compared to the control (Fig. 3E). Taken together this led us to hypothesize that IRF7 functions as a transcription activator of NMI. Using ChIP analysis, we determined that Teniposide treatment significantly enhanced IRF7 occupancy at position −28 in the NMI promoter (Fig. 3F). Finally, knockdown of IRF7 in breast cancer cells significantly diminished the ability of Teniposide to induce NMI following Teniposide treatment (Fig. 3G). Cumulatively our observations confirm that Teniposide upregulates NMI transcription through induction of IRF7.

### Teniposide-induced NMI modulates EMT

**Fig. 4:**
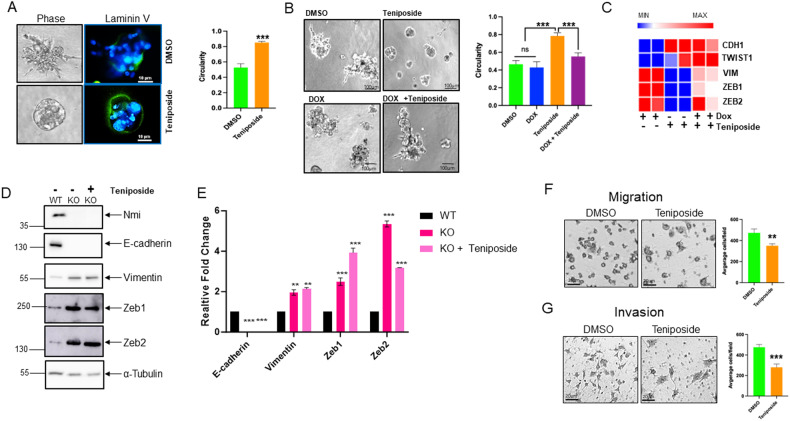
Teniposide-induced NMI modulates EMT. **A** Representative images of 1833 cells grown in 3D culture with Teniposide (500 nM) containing media. Phase images highlight dramatic changes in cell morphology. Adjacent color photomicrographs depict images of 3D cultures stained for basement membrane protein Laminin V (green) and with DAPI (blue), scale bars = 10 µm. Graph represents quantification of circular morphology of 3D structures using NIS Elements analysis software. Circularity was measured in 5 spheroids/field across 10 random fields. **B** Photomicrographs of bright field imaging of 3D growth of 1833 cells with DOX inducible NMI–shRNA treated with Teniposide. Cells were cultured in 3D in 500 nM Teniposide or 2 µg/mL doxycycline, individually or simultaneously. Graph represents quantification of circularity of the 3D colonies using NIS Elements analysis software. Circularity was measured in 5 spheroids/field across 10 random fields. **C** Heat map of CDH1, TWIST1, VIM, ZEB1, and ZEB2 from focused RT-PCR array analysis (Supplementary Fig. 1A) with DOX inducible NMI–shRNA expressing cells treated with DOX alone or treated with DOX and Teniposide. Representative of two independent runs. **D** Protein lysate from cell line derived from Nmi-knockout (KO) tumors, cultured with or without Teniposide and from cell line derived from NMI wild-type tumors (WT) were probed for levels of E-cadherin, vimentin, Zeb1, and Zeb2. Tubulin levels were monitored as a loading control. **E** Bar graph representing qRT-PCR quantitation of levels of E-cadherin, Vimentin, Zeb1, and Zeb2 from cell line derived from Nmi-knockout (KO) tumors, cultured with or without Teniposide and from cell line derived from NMI wild- type tumors (WT). Data are presented as mean + SEM. *p* determined by Student’s *t* tests. **p < 0.01, ***p < 0.001. **F** MDA-MB-231 cells, pretreated for 24 h with vehicle control or 500 nM Teniposide were analyzed (4 h) for migration in serum-containing media with vehicle control or Teniposide. Cells were fixed and visualized with crystal violet staining. Cells from four random fields/well in three independent wells were counted. **G** Invasive ability of MDA-MB-231 cells pretreated as before was estimated using media with vehicle control or 500 nM Teniposide (16 h). Cells were fixed and visualized with crystal violet. For both (**F**) & (**G**), images were captured using a Nikon Eclipse Ti-U, and cells were quantified using ImageJ. Cells from four random fields/well in three independent wells were counted. The quantification is represented in adjacent bar graphs. **p < 0.01, ***p < 0.001.

To determine the functional consequences of low concentration of Teniposide, we pursued the evidence that compromised expression of Nmi stimulated an EMT program (Fig. 1E, F, and G). We explored if low concentration of Teniposide would affect attributes of EMT. We used a highly metastatic clonal variant of MDA-MB-231 (the 1833 cell line) that metastasizes to bone [19]. The 1833 cells cultured in 3D spheroid culture conditions showed a noteworthy response to Teniposide. Control cells displayed multiple invasive cellular projections (stellate-like morphology) along with a highly deformed and fragmented basement membrane. This is consistent with their metastatic behavior. Notably, Teniposide modulated the 3D growth morphology of these cells to a well-circumscribed spheroidal structure (circularity > 0.8) along with an intact basement membrane (Fig. 4A).

We then generated 1833 cells containing inducible shRNA against NMI. Thus, doxycycline (DOX) could be used to silence NMI expression induced by Teniposide. This tool allowed us to verify the relevance of NMI in mediating the effect of Teniposide. We then cultured 1833 cells in 3D with or without DOX in the presence or absence of Teniposide. Like their parental MDA-MB-231 cells, 1833 cells have very low expression of NMI and display highly mesenchymal-like invasive stellate growth. We observed that low-dose Teniposide alone had a vivid and statistically significant impact on the 3D morphology compared to the control. The colonies looked much rounded and compact. At base line, 1833 cells have very low levels of NMI, they are already highly mesenchymal. Thus, further NMI silencing expression (due to DOX-induced NMI–shRNA), did not show any noticeable gain of mesenchymal attributes. Importantly, DOX-induced silencing of NMI severely limited the ability of Teniposide to diminish the highly invasive 3D growth morphology. Measurements of circularity of the colonies demonstrate that even in presence of Teniposide, due to the silencing of NMI, the 3D growth morphology remains very irregular (Fig. 4B). These observations clearly indicate a critical role for NMI in mediating Teniposide’s action and reducing mesenchymal-like growth morphology.

To further understand the molecular details underlying the activity of Teniposide on mitigating mesenchymal attributes, we undertook a targeted analysis of EMT-associated gene sets utilizing inducible silencing of NMI in combination with Teniposide. Consistent with the 3D growth morphology patterns observed in Fig. 4B, Teniposide treatment caused a shift in the EMT-associated gene expression pattern (Supplementary Fig. 2A). Specifically, our results revealed that in response to Teniposide the steady state transcript levels of EMT-driving transcription factors, ZEB1 and ZEB2, and the mesenchymal marker vimentin (VIM) were significantly reduced. Additionally, there was a substantial increase in the epithelial marker CDH1 (E-cadherin). Most importantly, ZEB1, ZEB2, and vimentin showed a considerable inverse trend (i.e., increase) upon DOX-induced silencing of NMI in the presence of Teniposide (Fig. 4C). Overall, our observations strongly implicate that NMI plays a role in Teniposide-mediated negative regulation of ZEB1 and ZEB2. For reasons unclear to us, TWIST1 expression showed a slight increase with Teniposide. However, this increase was not sensitive to DOX-induced silencing of NMI expression and did not contribute to the phenotypic trends observed in Fig. 4A, B.

As represented in our schematic Fig. 1C, we established a tumor-derived cell line (KO) from the NmiKO mouse and a corresponding control cell line (WT) from the NmiWT mouse. The KO cells were remarkably fibroblastic and mesenchymal-like in their appearance compared to the WT cells (Supplementary Fig. 2B). Consistent with their mesenchymal-like morphologic characteristics, the KO cells lacked E-cadherin expression concomitant with a gain of vimentin, ZEB1, and ZEB2. Our immunoblot analysis reveals that Teniposide is unable to reverse the increased expression of these markers in the KO cells (Fig. 4D and corresponding qRT-PCR analysis represented in Fig. 4E). These observations once again endorse the involvement of NMI in the activity of Teniposide.

To further assess the non-invasive phenotype imparted upon invasive breast cancer cells by low concentrations of Teniposide, we measured migration and invasion abilities using transwell assay and motility using wound healing assay. The results clearly demonstrate that Teniposide significantly hinders the migration, invasion as well as motility (Fig. 4F, G and Supplementary Fig. 2C). Another DNA/RNA synthesis inhibitor from our screen, gemcitabine, also upregulated NMI expression (albeit to a lesser extent). In contrast to Teniposide, gemcitabine failed to demonstrate a robust negative effect on expression of the mesenchymal marker genes (Supplementary Fig. 1D).

### ZEB2 is a regulator of rRNA synthesis

**Fig. 5:**
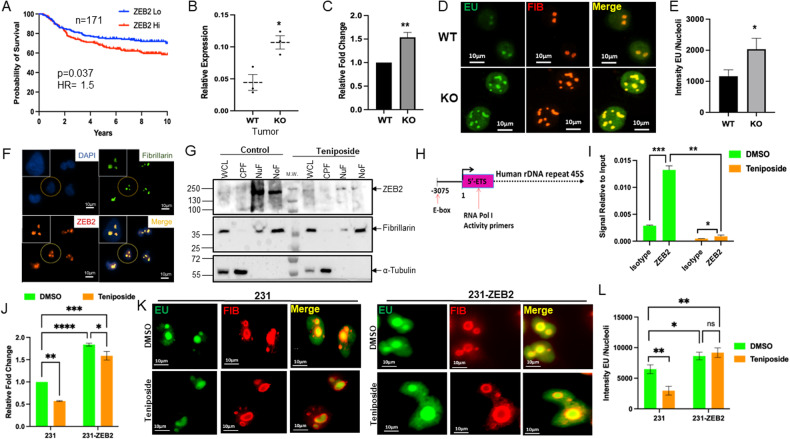
ZEB2 is a regulator of rRNA synthesis. **A** RNA expression data representing distant metastases free survival probabilities for breast cancer patients with high versus low-ZEB2 expression, *n* = 171 and *p* = 0.037, hazard ratio (log-rank) = 1.5. **B** Real-time PCR estimation of ITS-1 levels from RNA derived from Nmi-knockout (KO) versus wild-type (WT) tumors. **C** Real-time PCR estimation of ITS-1 levels from RNA derived from Nmi-knockout (KO) versus wild-type (WT) tumor cell lines. **D** Representative photomicrographs of EU incorporation to measure nascent rRNA transcripts in Nmi-knockout tumor cell lines. EU (green) and fibrillarin (red) merged images display EU incorporation in nucleolar regions. Intensity of EU incorporation per nucleoli quantified with NIS Elements. **E** Graph representing intensity of EU signal/nucleus. EU was measured in 20 cells/field across eight random fields. **F** Representative photomicrographs of sub-nuclear localization of ZEB2 using immunocytochemistry in MDA-MB-231 cells. Individual images show blue (DAPI) colored nuclei with green (fibrillarin) nucleoli and ZEB2 (red). **G** Western blot of total cell lysates (WCL), cytoplasmic fractions (CPF), Nuclear fractions (NuF), and nucleolar fractions (NoF) from control or Teniposide-treated MDA-MB-231 cells. Fibrillarin and α-tubulin are used for confirming enrichment of specific fractions. M.W. indicates molecular weight markers. **H** Human 45S rDNA repeat with E-box location marked at −3075. RNA Pol I activity primers are located in the 5′-ETS regions of the rDNA repeat. **I** ChIP analysis of ZEB2 (MDA-MB-231 cells) bound to the putative E-box site in the 47S rDNA after Teniposide treatment. Signal relative to 2% input for isotype and ZEB2. ZEB2 enriched at rDNA in native conditions as determined by significant enrichment of ZEB2 as compared to isotype. **J** Real-time quantitative PCR estimation of 5′-ETS levels in cells with or without overexpression of ZEB2, treated with control or Teniposide. **K** Representative photomicrographs of EU incorporation to measure nascent rRNA transcripts in MDA-MB-231 control and ZEB2 expressing cell lines treated with control or Teniposide. EU (green) and fibrillarin (red) merged images display EU incorporation in nucleolar regions. Intensity of EU incorporation per nucleoli quantified with NIS Elements. **L** Graph representing intensity of EU signal/nucleus. EU was measured across eight random fields. Data are presented as mean + SEM. *p* determined by Student’s *t* tests. *p < 0.05, **p < 0.01, ***p < 0.001, ****P < 0.0001.

A few studies have implicated a prognostic role for EMT-related transcription factors ZEB1 and ZEB2 in tumor types, such as ovarian, colorectal, and breast carcinomas [37–40]. Using the same cohort of patient data that we queried for NMI expression in Fig. 1B, we analyzed survival probabilities of patients in the context of ZEB1 or ZEB2 expression. ZEB1 expression did not demonstrate any significant impact on patient survival (Supplementary Fig. 3A). However, when stratified by ZEB2 expression in the breast tumor, patients with high-ZEB2 levels demonstrated significantly lower survival probability than those with low-ZEB2 levels, with a median survival of 4267 days in low-ZEB2 expressors in contrast to 2965 days in high-ZEB2 patients (Fig. 5A).

A recent study by Prakash et al. uncovered a novel association between EMT and ribosome biogenesis. That study highlighted that the induction of ribosome biogenesis is an integral feature of the EMT program [41]. We analyzed RNA-seq data from the NmiKO mammary tumors (Fig. 1D) and observed a significant enrichment of gene sets focused on ribosome biogenesis (Supplementary Fig. 3B). To further validate this observation, we quantified the levels short-lived internally transcribed spacer (ITS) region of the 47S rRNA (murine) by quantitative RT-PCR using RNA from the tumors (Fig. 5B and Supplementary Fig. 3C) as well as RNA from tumor-derived cell lines (WT and KO) (Fig. 5C). In both cases, we observed a clear increase in levels of ITS indicative of increased activity of RNA Pol I in absence of Nmi expression. To independently verify this observation, we evaluated the nascent rRNA transcript abundance in tumor-derived cell lines (WT and KO) by EU incorporation assay [26] (Fig. 5D). Compared to the WT cells, the KO cells showed more numerous nucleoli per nucleus, indicative of increased ribosome biogenesis [42, 43]. These nucleoli displayed intense EU incorporation (twice as intense) compared to the Nmi wild-type cell line, confirming increased nascent rRNA synthesis (Fig. 5E).

Since ZEB2 was downregulated by Teniposide (Fig. 4C, D, and E), we hypothesized that ZEB2 may be a critical regulator of ribosome biogenesis. Ribosome biogenesis is a complex and involved process that is critically dependent on rRNA transcription in the nucleolus [44, 45]. Thus, we aimed to determine the intra-cellular localization of ZEB2. Immunofluorescent imaging revealed that ZEB2 is prominently localized to the nucleolus. This was evidenced by an impeccable overlay of ZEB2 with fibrillarin, a well-known marker of the nucleolus (Fig. 5F). To further verify these observations, we isolated nucleoli from MDA-MB-231 breast cancer cells treated with Teniposide or vehicle control. Western blot analysis of the nucleolar proteins revealed that ZEB2 is enriched in the nucleolar fraction (Fig. 5G). It is also evident that Teniposide significantly reduces ZEB2 levels in the nucleoli to <5% of that of control (Fig. 5G and respective quantitation in Supplementary Fig. 3D). We explored the possibility that ZEB2 may bind to rDNA loci. Examination of rDNA promoter revealed a highly conserved E-box consensus sequence at −3075 position (Fig. 5H). ChIP analysis confirmed ZEB2 occupancy at rDNA spanning the E-box (Fig. 5I). We also observed that low-dose Teniposide dramatically reduced ZEB2 occupancy at this E-box (Fig. 5I). To evaluate the impact of low concentration of Teniposide on rRNA transcription, we analyzed the short-lived 5′-ETS region of the 47S rRNA (human) by real-time PCR. Teniposide treatment of MDA-MB-231 cells significantly reduced the abundance of the 5′-ETS, indicating that rDNA transcription is compromised by Teniposide (Fig. 5J). To further investigate the involvement of ZEB2 in regulating rDNA transcription, we exogenously overexpressed ZEB2 in MDA-MB-231 cells (Supplementary Fig. 3E). Using qRT-PCR measurement of the 5′-ETS, we observed that ectopic expression of ZEB2 prompted hyperactivation of rRNA transcriptional rates; moreover, ZEB2 overexpression circumvented low-dose Teniposide-induced repression of rRNA transcription in MDA-MB-231. (Fig. 5J). EU incorporation into nascent rRNA transcripts confirmed the increased rRNA production resultant from exogenous ZEB2 expression; furthermore, ZEB2 blunted the impact of Teniposide on nascent rRNA abundance (Fig. 5K, L) Conversely, silencing ZEB2 expression using shRNA significantly reduced the abundance of the 5′-ETS fragments, indicating compromised rDNA transcription (Supplementary Fig. 3F, G). Overall, our observations reveal that both Teniposide and NMI negatively regulate rRNA biogenesis and that ZEB2 is a responsible regulator of rDNA transcription.

### Teniposide reduces metastasis

**Fig. 6:**
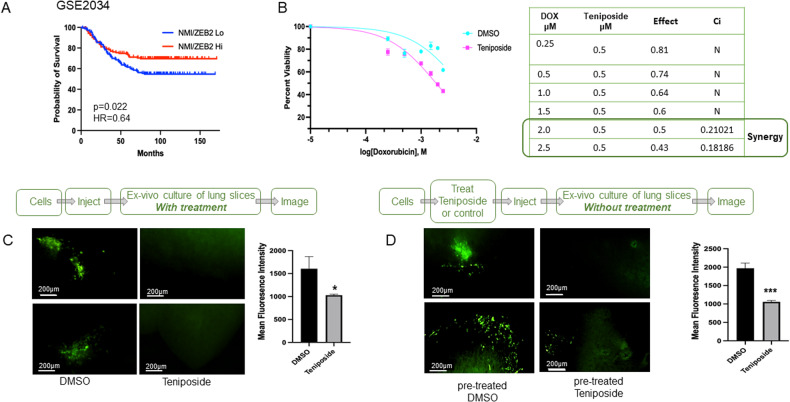
Teniposide reduces metastasis. **A** Kaplan–Meier survival analysis using gene expression ratio of NMI/ZEB2 using breast cancer patient tumor transcriptomic analysis (GSE2034) (*p* = 0.02). **B** Estimation of viability of MDA-MB-231 cells treated with Doxorubicin, with DMSO or Teniposide. MDA-MB-231 cells were treated with varying concentrations of Doxorubicin alone or a combination of Doxorubicin and 0.5 µM Teniposide. The combination index was calculated using CompuSyn software V 1.0. synergistic (Ci < 1), antagonistic (Ci > 1), or no effect (Ci = 0). “*N*” indicates no synergy. **C** PuMA using GFP-labeled 1833 cells. Lung sections were treated with 500 nM Teniposide or control. Images were taken on day 21 of culture. Mean fluorescence intensity of colonies was quantified (adjacent Bar graph) with NIS Elements (*n* = 6). **D** PuMA using GFP-labeled 1833 cells pretreated (for 48 h prior to injection) with 500 nM Teniposide or control. Lung sections were maintained in media without any drugs for 21 days. Mean fluorescence intensity of colonies was quantified (adjacent Bar graph) with NIS Elements (*n* = 6). Data are presented as mean + SEM. *p* determined by Student’s *t* tests. *p < 0.05, ***p < 0.001.

A few studies have implicated a prognostic role for ZEB1 and ZEB2 in tumor types such as ovarian, colorectal, and breast carcinomas [37–40]. Using the online tool (http://kmplot.com/analysis/) [31, 46] we analyzed survival probabilities in patients with high or low ZEB1 or ZEB2 expression using the publicly available dataset GSE2034 [47]. ZEB1 levels did not seem to significantly impact patient survival (Supplementary Fig. 3A). Extending our investigation to include patient survival in the context of NMI and ZEB2 gene expression, we analyzed the gene expression ratio of NMI/ZEB2. Kaplan–Meier survival analysis showed that breast cancer patients with low ratio of NMI/ZEB2 (NMI/ZEB2 Lo) had significantly worse (*p* = 0.02) relapse-free survival compared to patients with a higher ratio (Fig. 6A). These data support that the relative expression of NMI and ZEB2 could be an important determinant of disease progression.

Patient outcome is adversely impacted by drug-resistant relapse and metastasis [48, 49]. EMT has been credited for promoting a drug-resistant phenotype [50–52]. Thus, we tested if Teniposide will enhance the sensitivity of MDA-MB-231 cells to Doxorubicin, a standard-of-care drug for breast cancer. Our results showed that co-treatment with low-dose Teniposide and Doxorubicin synergistically decreased the viability of these cells (Fig. 6B). Finally, to test if Teniposide may affect metastasis, we used a PuMA. MDA-MB-231-1833 breast cancer cells were injected via tail vein in athymic nude mice, lungs were inflated, perfused with agarose, sectioned, and then kept in culture to monitor the outgrowth of metastasis. While the vehicle control lungs showed a remarkable metastatic outgrowth, sections of lungs incubated in Teniposide-containing media failed to form viable metastatic colonies as visualized by the fluorescence of GFP-expressing cells (Fig. 6C). To specifically address the negative impact of Teniposide on the invasive program of EMT, we pretreated the cells with Teniposide for 24 h prior to injecting into mice for PuMA assay. The assay was conducted as done previously, with the exception that, lung sections were cultured without Teniposide (Fig. 6D). Pretreated cells were severely limited for their ability to form metastatic colonies. Since the tumor cells were subjected to Teniposide only prior to the injection, our observations suggest that Teniposide-treated tumor cells failed to invade the lung parenchyma.

## Discussion

Metastatic colonization of breast cancer requires a precisely coordinated series of events that allow cells to leave the primary tumor and invade at the metastatic site. EMT is one of the well-studied events of metastatic progression [3, 7, 10, 53]. Studies have long implicated that acquisition of mesenchymal plasticity by tumor cells following erroneous onset of developmental signaling culminates into a myriad of phenotypic changes such as stemness, cell motility, and drug resistance [1, 52, 54]. However, EMT-targeted therapeutic intervention has remained quite elusive [55–57]. NMI is critically important in restricting breast cancer metastasis [3, 7, 10] and is a potent regulator of EMT [17, 18, 58]. Thus, upregulation of NMI expression is a promising approach. As such, we sought to upregulate NMI expression to curb the mesenchymal phenotype. For this, we conducted an unbiased assessment of FDA-approved compounds for upregulating NMI expression. The most potent drivers of NMI expression were topoisomerase inhibitors; Teniposide stood out as a compound with the most consistent effect.

Teniposide is a semisynthetic derivative of podophyllotoxin that acts to inhibit DNA synthesis by forming a complex with DNA and induces breaks in double-stranded DNA. Its known activity at the cytotoxic dose is that it binds to topoisomerase II and prevents DNA repair thereby allowing for accumulation of DNA breaks. These breaks prevent cells from entering the mitotic phase of the cell cycle and lead to cell death [59]. In this current study, a very low dose of Teniposide presented as the most robust mediator of NMI expression, leading to significant changes in the EMT program, mediated by an IRF7-dependent mechanism. At nanomolar concentrations, Teniposide showed previously unreported effects on EMT and ribosome biogenesis that appear to be independent of its cytotoxicity.

Our work highlighted ZEB2 as a key node in enabling an EMT programmatic shift. ZEB2 is an established transcriptional regulator of the EMT program that spurs malignant/invasive cellular attributes. ZEB2 is classically described as an important transcriptional repressor, exerting its effect on EMT via downregulation of E-cadherin, an effect that we consistently noticed (e.g., Fig. 4C, D, and E). Emerging reports have begun to uncover the importance of ZEB2 in many EMT-driven processes such as drug resistance, stemness, apoptosis, recurrence, and metastasis [60, 61]. Even though ZEB2 is predominantly perceived as a transcriptional repressor, a number of reports have also identified its role as a transcriptional activator, mostly in the context of cancer. In particular, studies identified cooperation between ZEB2 and SP1 leading to transcriptional activation of vimentin, integrin α5, and cadherin 11. Furthermore, survivin, Bcl-2, cyclin D1, and VEGF were also found to be transcriptionally activated leading to enhanced cell survival, angiogenesis, and cancer progression [62, 63]. The clinical relevance of ZEB2 as a potential biomarker has been underscored in both, ovarian and colorectal cancers, wherein elevated ZEB2 correlated with poor prognosis, recurrence, and metastasis [40, 64]. Limited studies of ZEB2 are available in breast cancer patient cohorts, with one study identifying increased ZEB2 in triple-negative breast cancer as a prognostic indicator for poor survival [65]. Our survey of TCGA breast cancer patient data for ZEB2 further highlights the importance of ZEB2 in predicting patient survival. Accentuating the importance of ZEB2 was the inverse correlation between NMI and ZEB2 expression, wherein low NMI/ZEB2 ratio yielded unfavorable survival probability. Teniposide treatment of breast cancer cells yielded high NMI/ZEB2 expression that correlated with a dramatic shift in their EMT phenotype. In summary, we discovered a previously unidentified role for ZEB2 in regulating rDNA transcription, demonstrating that ZEB2 binds rDNA promoter and activates rRNA biogenesis and Teniposide negatively impacts this process by decreasing the expression of ZEB2.

Recent reports have suggested an interplay between EMT and ribosome biogenesis [66]. A hallmark study by Prakash et al. described an association between initiation of the EMT program concomitant with activation of rDNA transcription, in which inhibition of rRNA synthesis obstructed the EMT program and reduced metastasis [41]. The exact causal relationship between ribosome biogenesis and EMT remains elusive; for example, a recent study described upregulation of La-related protein 6 by EMT as a mediator of localization of ribosomal proteins, suggesting regulation of ribosome biogenesis downstream of EMT [67]. It is certainly feasible that EMT and ribosome biogenesis may crosstalk with each other. It is well known that ribosome biogenesis is an important event for metabolically active cells, and it is a logically appealing thought that increased ribosome biogenesis may be essential for the execution of the metabolic plasticity that is required for the EMT program.

**Fig. 7:**
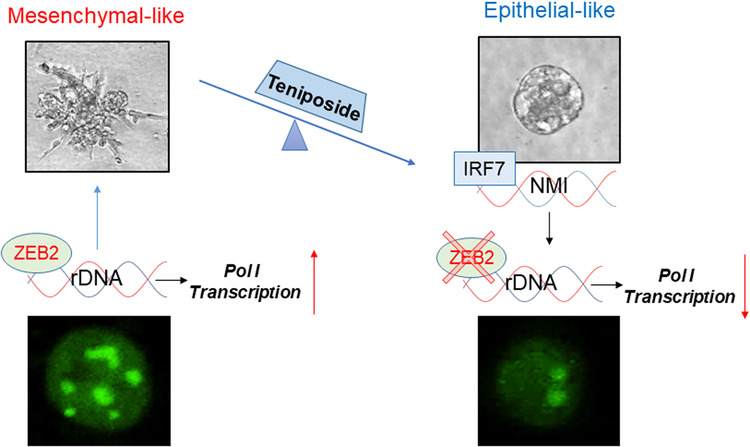
Schematic summary of findings. Mesenchymal transcription factor ZEB2 is in the nucleolus at the rDNA and elevates rDNA transcription that supports mesenchymal phenotype. Low-dose Teniposide promotes epithelial-like phenotype by inducing IRF7-driven NMI expression that decreases ZEB2 levels significantly and consequently RNA Pol I transcription activity.

Our previous studies reported that NMI is a potential pivotal node to restrict mesenchymal transition both, in mammary development and in cancer models [16, 17]. Recently, loss of NMI was highlighted as an important event that shapes rRNA methylation in hypoxia, eliciting a translational switch [25]. Teniposide upregulates NMI, and in turn, induces an epithelial-like phenotype concomitant with downregulation of RNA Pol I transcription. However, details of ZEB2 activation of rRNA transcription require further investigation. It may be that ZEB2 is a direct transcriptional activator of rRNA transcription or works in concert with other RNA Pol I regulatory elements such as UBTF or members of the SL1 complex to regulate rRNA biogenesis during the EMT process. Overall, our current study underscores the link between ribosome biogenesis and EMT (Fig. 7).

EMT is linked to a number of cancer hallmarks, particularly invasion, metastasis, survival, stemness and drug resistance, and more recently ribosome biogenesis [2, 11, 41, 53]. Several landmark studies have linked increased EMT signature with poor survival [68–70]. Thus, it is of paramount importance to uncover novel treatment modalities that target the EMT program. Teniposide is currently used as an effective treatment in non-Hodgkin’s lymphoma and acute lymphocytic leukemia, as well as other cancers [59, 71]. In most cases, a higher concentration of Teniposide is used to leverage its DNA-damaging properties as a topoisomerase inhibitor [59]. However, other modes of action have also been described. Historically Teniposide has been tested in breast cancer, but the concentration range used for treatment was much higher and the trial results were not promising [72, 73]. Our current study reveals a promising role of Teniposide at low dose in reducing metastatic seeding in-part through blocking EMT. Ribosome biogenesis, specifically inhibition of RNA Pol I activity, is an up-and-coming cancer drug target due to the expectation that normal cells would be spared as they are less reliant on RNA Pol I activity than cancer cells. Inhibitors of RNA Pol I activity such as CX-5467, BMH-21, and metarrestin are at various stages of development [74–77]. Some reports in the literature have suggested the potential of topoisomerase inhibitors to influence EMT. In one such study, neoamphimedine, previously discovered as a TOP2A inhibitor, was identified as a potent EMT inhibitor based on significant downregulation of vimentin. Furthermore, neoamphimedine was identified to act as a competitive ATP inhibitor to block topoisomerase function [78]. In addition, that study highlighted the ability of topoisomerase IIα to act as a TCF binding partner, thereby promoting an EMT-driven malignant phenotype. A follow-up study identified novel inhibitors of TOP2A that reverted EMT phenotype in a TCF-dependent manner [79]. In this context, it is important to note that some reports indicate that at pharmacologically used concentrations, CX-5461 targets topoisomerase II beta (TOP2B), not RNA Pol I and influences topoisomerase-related DNA damage response [80–82].

These perspectives make our evidence demonstrating the effectiveness of low dose of Teniposide on impeding RNA Pol I activity very attractive. Teniposide is FDA approved for the treatment of refractory childhood acute lymphoblastic leukemia, non-Hodgkin lymphoma, and other types of cancers and usually is combined with other chemotherapeutic agents. Its use (at the clinically used dose) results in severe bone marrow suppression, gastrointestinal toxicity, hypersensitivity reactions, and alopecia. Our work presents that Teniposide at low concentration, is an effective modulator of the EMT program. Specifically, Teniposide works through an IRF7–NMI–ZEB2 axis to curb EMT. We show that ZEB2 is a positive regulator of RNA Pol I transcriptional activity and rRNA biogenesis. Through limiting ZEB2 expression, Teniposide had a remarkable inhibitory effect on RNA Pol I and rRNA biogenesis. Furthermore, Teniposide markedly reduced pulmonary colonization of breast cancer cells.

## Conclusions

At the low dose that we investigated, which is a fraction of the clinically employed dose, we registered a remarkably reduced lung colonization and invasion. Importantly at such a low dose, Teniposide enhanced the sensitivity of breast cancer cells to Doxorubicin, a chemotherapeutic routinely administered to breast cancer patients. These observations coupled with its ability to impede EMT and target rRNA biogenesis make Teniposide a viable candidate to be repurposed in combination with current standard-of-care treatment for breast cancer as a therapeutic option to decrease breast cancer metastases.

### Limitation

Ribosome biogenesis may be affected by topoisomerase inhibition; however, the degree to which topoisomerase poisons can affect ribosome biogenesis in different solid tumors and other hematological malignancies is difficult to establish [83]. Thus, more work is needed to investigate if ribosome biogenesis is impacted by topoisomerase inhibition and the cellular context.

The known mechanism of action of Teniposide as a topo-poison involves a dose-dependent single/double-stranded DNA break. The cytotoxic effects of Teniposide are related to the extent of double-stranded DNA breaks produced in cells, which is dependent on its concentration. At nano-molar concentrations (used in our study) Teniposide does not inflict substantial DNA damage. We also note that in our study Teniposide activity is not via the DNA damage response mediated by p53. Our screen was performed in MDA-MB-231 cells which are reported to bear a mutant P53. Additionally, MDA-MB-453 breast cancer cells that are practically devoid of p53 protein showed a similar response to Teniposide as MDA-MB-231, in activating the NMI-619 luciferase reporter, NMI and IRF7 transcript levels (Supplementary Fig. 4A, B, and C). Teniposide is reported to induce IFN-I and NF-κB signaling via the cGAS-STING activation [84, 85]. Thus, we have assessed the impact of Teniposide on NF-κB activation in both MDA-MB-231 and MDA-MB-453. Teniposide induced a robust activation of the NF-κB pathway as seen by phospho-P65 in both cell lines, albeit with a more pronounced effect in MDA-MB-231 (Supplementary Fig. 4C). Cumulatively, we propose that mechanism of action of Tenoposide is independent of P53 and likely through NF-κB.

### Supplementary information

Supplementary Materials

Full length Blots

## Data Availability

Details of all data generated or analyzed during this study are included in this article and its Supplementary files.
