# Supplementary material for: Targeting EMT using low-dose Teniposide by downregulating ZEB2-driven activation of RNA polymerase I in breast cancer
Source: Cell Death Dis. 2024 May 8;15(5):322. doi: 10.1038/s41419-024-06694-7 (PMC11079014; doi:10.1038/s41419-024-06694-7)
Supplement: Supplementary file 1 — Supplementary Materials [file 41419_2024_6694_MOESM1_ESM.pdf]

A.

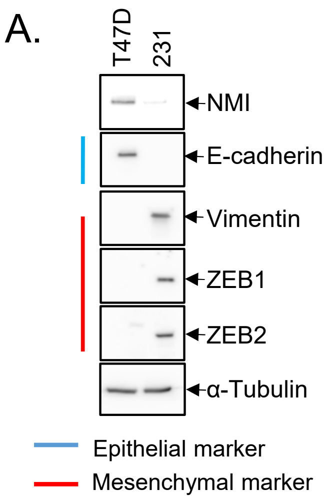

B.

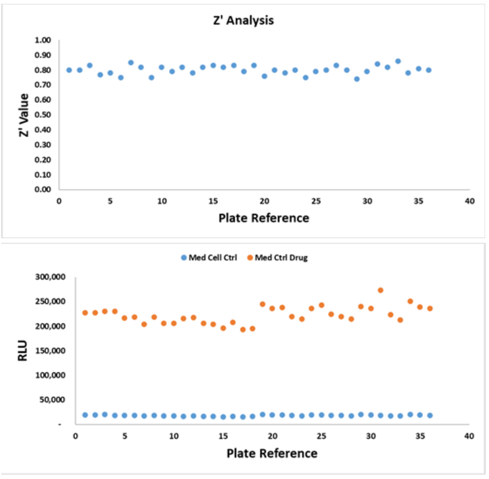

D.

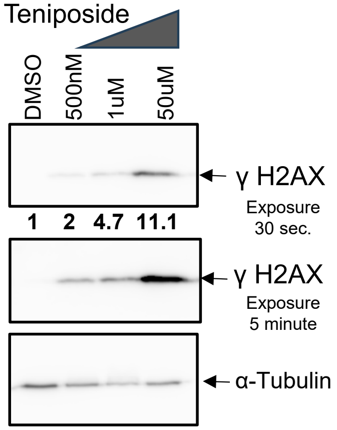

F.

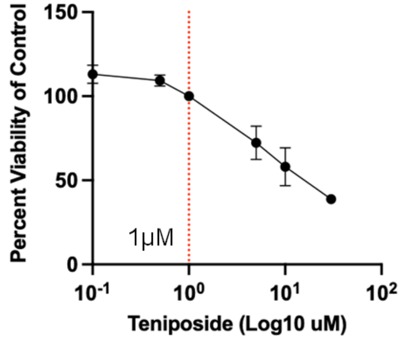

C.

Target	Drug Name	Supplier	Supplier ID	RLU (Run 1)	Fold Increase (Run 1)	RLU (Run 2)	Fold Increase (Run2)
DNA/RNA Synthesis	Cladribine, Leustatin	Selleck	S1199	41960	2.32	37200	1.95
DNA/RNA Synthesis	Clofarabine, Clolar, Evoltra	Enzo	DL-103	53320	2.77	53280	2.74
DNA/RNA Synthesis	Clofarabine, Clolar, Evoltra	Selleck	S1218	46120	2.3	50920	2.6
DNA/RNA Synthesis,	Gemcitabine HCl, Gemzar	Selleck	S1714	46920	2.34	48520	2.48
DNA/RNA Synthesis,	Gemcitabine HCl, Gemzar	Enzo	DL-215	46120	2.43	45840	2.25
DNA/RNA Synthesis,	Gemcitabine HCl, Gemzar	Selleck	S1149	50320	2.51	41720	2.13
EGFR, Topoisomerase	Genistein	Selleck	S1342	51040	2.55	50520	2.58
EGFR, Topoisomerase	Genistein, Fosteum	MicroSource	00210296	48560	2.86	59520	2.96
Topoisomerase	Irinotecan HCl Trihydrate, Campto, CPT 11, Camptosar, Irinotecan	Selleck	S2217	80600	4.3	86080	4.68
Topoisomerase	Irinotecan Hydrochloride, Camptosar	MicroSource	01505821	46240	2.67	51440	2.92
Topoisomerase	Irinotecan, Camptosar, Campto	Selleck	S1198	76640	3.82	84680	4.32
Topoisomerase	Etoposide	MicroSource	01500903	42240	2.33	52640	2.84
Topoisomerase	Etoposide	Enzo	GR-307	53280	2.8	58040	2.85
Topoisomerase	Etoposide	Selleck	S1225	56560	2.82	52920	2.7
Topoisomerase	Etoposide, VP-16, Vepesid	AACF	414623	39880	2.43	42800	2.13
Topoisomerase	Camptothecin	Selleck	S1288	122440	6.11	129800	6.63
Topoisomerase	Teniposide, Vumon	MicroSource	01504094	61600	3.54	71160	3.94
Topoisomerase	Teniposide, Vumon, VM-26, Vehem, NSC 122819	Selleck	S1787	60400	3.22	61720	3.36
Topoisomerase	Topotecan·HCl	Enzo	DL-518	62720	3.26	68400	3.52

E.

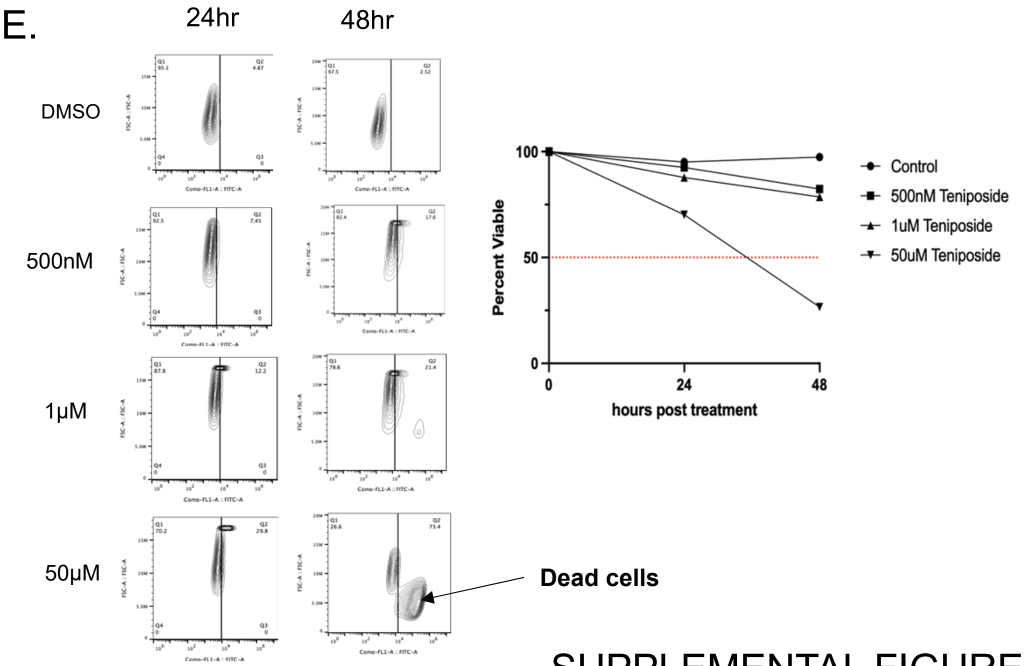

SUPPLEMENTAL FIGURE 1

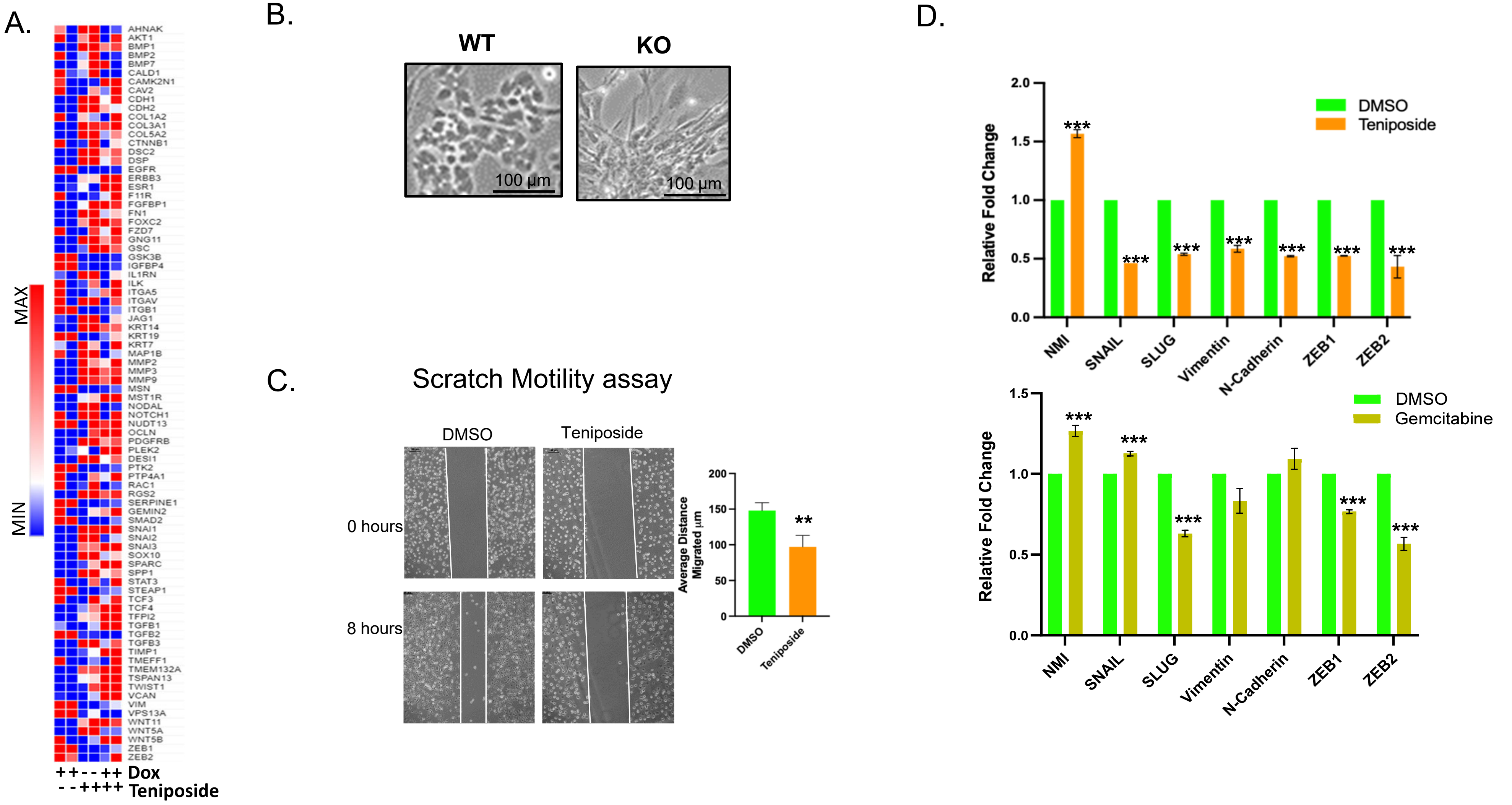

SUPPLEMENTAL FIGURE 2

A.

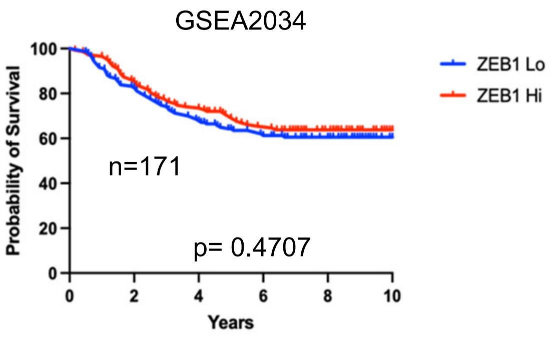

B.

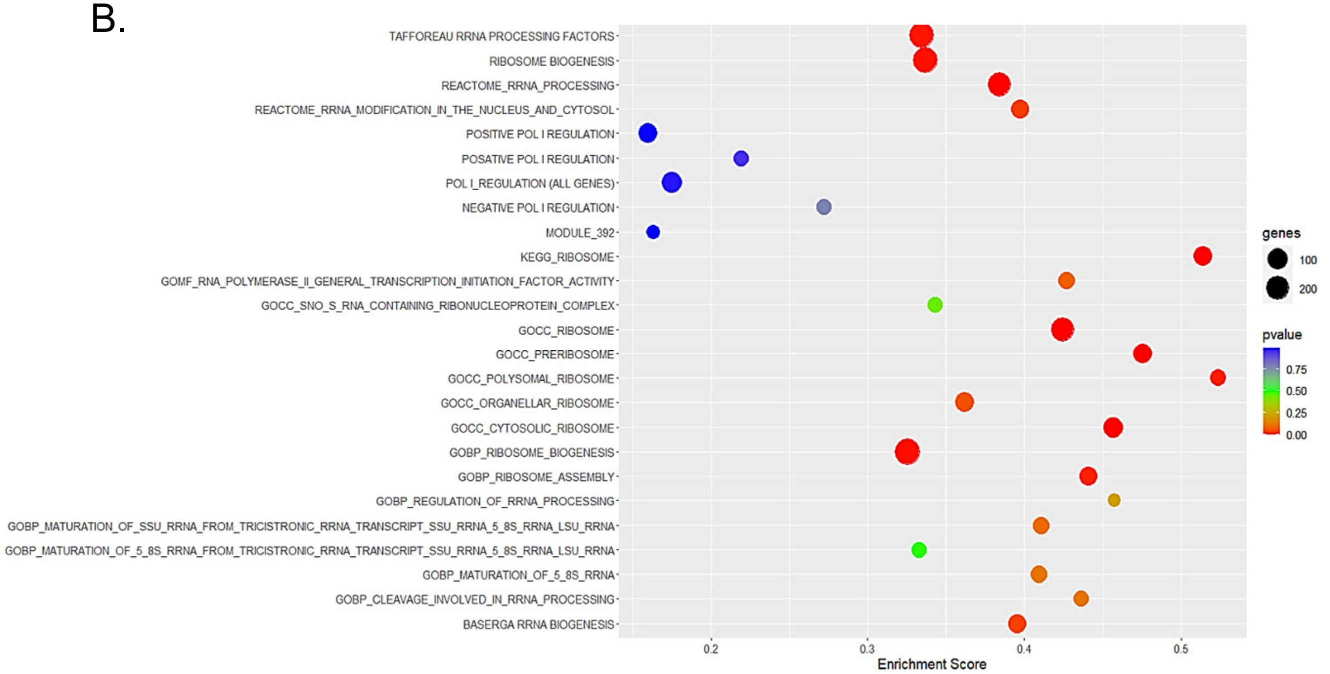

C.

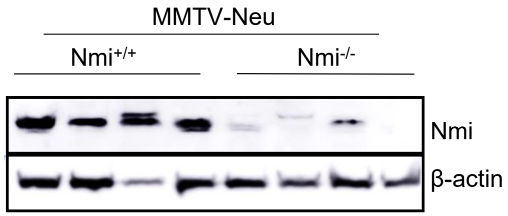

D.

	Teniposide/ Control Nuclear Fraction	Teniposide/ Control Nucleolar Fraction
ZEB2	0.061	0.033
ZEB2 (Normalized to FBL)	0.18	0.038

E.

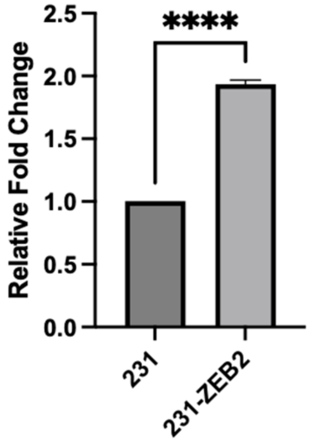

F.

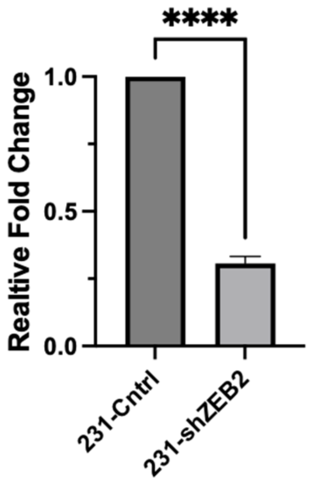

G.

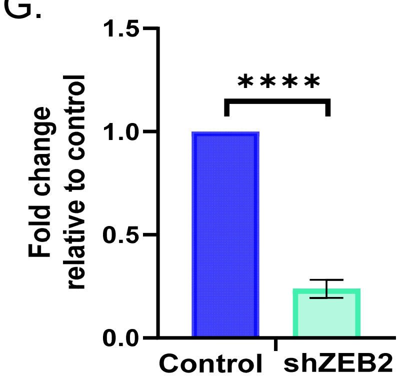

SUPPLEMENTAL FIGURE 3

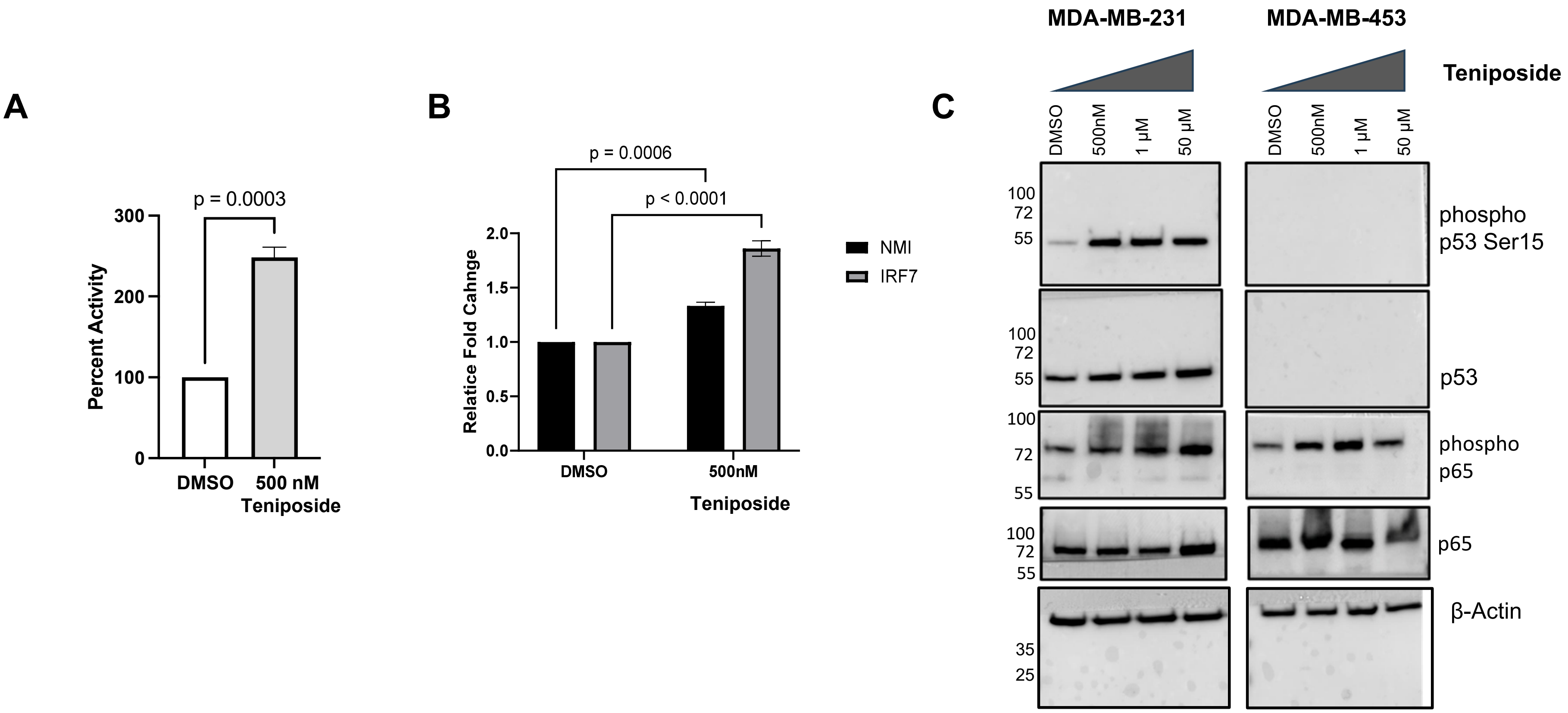

SUPPLEMENTAL FIGURE 4

Supplemental Figure Legends

Supplemental Figure S1.

(A) Western blot analysis of T47D and MDA-MB-231 cells for EMT markers. **(B)** Z' analysis and raw data values (RLU) of assay plate controls, demonstrating reproducibility of assay in 200ng/mL IFNγ-treated cells. **(C)** Table of top hits from HTS of FDA compound library. Two major compound classes identified: DNA/RNA synthesis inhibitor or topoisomerase inhibitor. Raw data presented from duplicate runs along with fold change to control. **(D)** Western blot (Short 30 seconds and long 5 min. exposure) of γH2AX levels in MDA-MB-231 cells following Teniposide treatment. **(E)** Dose titration of Teniposide to nanomolar ranges to estimate effect on cell viability using MDA-MB-231. Cells were treated with 500nM, 1μM, 50μM Teniposide or vehicle control and cell assessed for viability using Zombie Green Fixable Viability Kit. **(F)** Dose titration of Teniposide to nanomolar ranges to estimate effect on cell viability. Cells treated for 24 hours and viability assed with CyQuant. Data presented as percent viable cells compared to DMSO control. Red line marks dosage used for subsequent studies.

Supplemental Figure S2.

(A) Complete heat map of gene expression from EMT signaling specific RT-PCR array analysis of cells with DOX inducible NMI-shRNA treated with Teniposide. Representative of two independent runs. **(B)** Morphology of cell lines derived from NMI wild type (WT) tumors compared with NMI-KO tumor (KO) when grown on tissue culture plates. **(C)** MDA-MB-231 cells (150,000) were seeded in 6-well plates and treated with vehicle control or 500nM Teniposide. After 24 hours scratch-wound was produced the center of each well. Distance migrated was measured using NIS Elements (Nikon). ***$p < 0.01$ **(D)** Real time PCR of various mesenchymal markers and NMI 24 hours after Teniposide or gemcitabine treatment. Graph represents relative fold change compared to DMSO control. Gemcitabine has minimal effect on expression of EMT markers. Data are presented as mean + SEM. p determined by multiple t test comparison. ***$p < 0.001$.

Supplemental Figure S3.

(A) TCGA patient data representing survival probabilities in breast cancer patients with high versus low ZEB1-expression; n=171 and p=0.721 **(B)** Bubble plot of GSEA of ribosome biogenesis gene sets using RNA-Seq data from NMI-WT and NMI-KO tumors. Circle diameter depicts relative number of genes in each associated gene set, and red indicates most significant enrichment score. **(C)** Western blot to confirm loss of Nmi protein in Nmi knockout tumors. **(D)** Table representing densitometry of ratios of band densities (Teniposide/control) from nuclear fraction or nucleolar fractions for ZEB2 and ZEB2 upon normalizing to fibrillarin. **(E)** Level of ZEB2 mRNA in MDA-MB-231 cells when transfected with control (Cntrl) of shZEB1 construct. **(F)** Level of ZEB2 transcript in control and cells with shRNA for ZEB2 **(G)** Silencing ZEB2 expression using (shZEB2) significantly reduced the abundance of the 5'-ETS fragments.

**** represents P< 0.0001

Supplemental Figure S4.

(A) Luciferase activity of NMI-619 luciferase reporter in MDA-MB-453 cells treated with Teniposide (500nM) or DMSO control. Experiment conducted in triplicate. **(B)** Total RNA from MDA-MB-453 cells treated with Teniposide (500nM) or DMSO control was analyzed for NMI and IRF7 transcript levels using quantitative RT-PCR. Experiment conducted in triplicate. **(C)** Western blot analysis of lysates from MDA-MB-231 and MDA-MB-453 following treatment with Teniposide at 500nM, 1μM, 50μM concentrations. Protein lysates were resolved on single membrane for P53, pSer15-p53, phosphor p65 and β-Actin. Lysate for p65 was resolved on a separate membrane. The images are spliced for visual depiction. A single β-actin control was used to determine loading.

Supplemental Table S1: Primer used in this study

**Name**	**Primer sequence (5'-3')**
To detect IRF7 occupancy at the NMI basal promoter elements	
NMI-IRF7-F	CGGAAACTTCAGGTATACTTC
NMI-IRF7-R	CTGCTTTAACGGCGATTTTTC
To detect ZEB2 occupancy at the rDNA	
Zeb2-rDNA-F	GTACTTTTAGTAGAGACGGTG
Zeb2-rDNA-R	CACTTTGGGAGGCTAAGGC
To detect steady state levels of 5'ETS (human)	
5'ETS 851-961-F	GAACGGTGGTGTGTCGTT
5'ETS 851-961-R	GCGTCTCGTCTCGTCTCACT
To detect steady state levels of Actin (human)	
Actin-F	CATGTACGTTGCTATCCAGGC
Actin-R	CTCCTTAATGTCACGCACGAT
To detect steady state levels of 45S ITS1 (murine)	
ITS1-F	CCGGCTTGCCCGATTT
ITS1-R	GGCCAGCAGGAACGA
To detect steady state levels of Actin (murine)	
Actin-F	GGCTGTATTCCCCTCCATCG
Actin-R	CCAGTTGGTAACAATGCCATGT

Supplemental Table S2: Eight unique compounds that were confirmed positive hits of the screen

Note: Many compounds had multiple vendors

Target	Drug Name	Supplier	Supplier ID	RLU (Run 1)	Fold Increase (Run 1)	RLU (Run 2)	Fold Increase (Run2)
DNA/RNA Synthesis	Cladribine, Leustatin	Selleck	S1199	41960	2.32	37200	1.95
DNA/RNA Synthesis	Clofarabine, Clolar, Evoltra	Enzo	DL-103	53320	2.77	53280	2.74
DNA/RNA Synthesis	Clofarabine, Clolar, Evoltra	Selleck	S1218	46120	2.3	50920	2.6
DNA/RNA Synthesis,	Gemcitabine HCl, Gemzar	Selleck	S1714	46920	2.34	48520	2.48
DNA/RNA Synthesis,	Gemcitabine HCl, Gemzar	Enzo	DL-215	46120	2.43	45840	2.25
DNA/RNA Synthesis,	Gemcitabine HCl, Gemzar	Selleck	S1149	50320	2.51	41720	2.13
EGFR, Topoisomerase	Genistein	Selleck	S1342	51040	2.55	50520	2.58
EGFR, Topoisomerase	Genistein, Fosteum	MicroSource	00210296	48560	2.86	59520	2.96
Topoisomerase	Irinotecan HCl Trihydrate, Campto, CPT 11, Camptosar, Irinotecan	Selleck	S2217	80600	4.3	86080	4.68
Topoisomerase	Irinotecan Hydrochloride, Camptosar	MicroSource	01505821	46240	2.67	51440	2.92
Topoisomerase	Irinotecan, Camptosar, Campto	Selleck	S1198	76640	3.82	84680	4.32
Topoisomerase	Etoposide	MicroSource	01500903	42240	2.33	52640	2.84
Topoisomerase	Etoposide	Enzo	GR-307	53280	2.8	58040	2.85
Topoisomerase	Etoposide	Selleck	S1225	56560	2.82	52920	2.7
Topoisomerase	Etoposide, VP-16, Vepesid	AACF	414623	39880	2.43	42800	2.13
Topoisomerase	Camptothecin	Selleck	S1288	122440	6.11	129800	6.63
Topoisomerase	Teniposide, Vumon	MicroSource	01504094	61600	3.54	71160	3.94
Topoisomerase	Teniposide, Vumon, VM-26, Vehem, NSC 122819	Selleck	S1787	60400	3.22	61720	3.36
Topoisomerase	Topotecan·HCl	Enzo	DL-518	62720	3.26	68400	3.52

Supplemental Table S3: BRM961a TMA clinicopathological characteristics

Characteristic	Total Number of samples	NMI staining IHC score
**ER receptor status**		
-	34	3.882353
+	18	4.666667
++	20	5.6
+++	23	4.565217
Unknown	42	
**PR receptor status**		
-	67	4.2
+	22	5.9
++	6	4.2
Unknown	42	
**HER2 receptor status**		
0	55	4.8
2+	10	4.1
3+	30	4.2
Unknown	42	
**Subtype**		
LUM-A	28	4.7
LUM-B	22	4.4
TNBC	16	3.8
HER2+	18	3.9
Unknown	42	
**Tissue type**		
Normal	12	6.2
DCIS	02	8
Primary tumor	46	4.6
LN metastases	36	3.9
**Grade**		
I	17	4.06
II	48	4.02
III	02	4
Unknown		

Characteristic	Number of Patients/samples	NMI staining IHC score
**ER receptor status**		
-	21	4.3
+	6	4.5
++	7	5.2
+++	14	4.8
Unknown	21	
**PR receptor status**		
-	36	4.6
+	8	5.1
++	3	4.3
Unknown	21	
**HER2 receptor status**		
0	25	4.9
2+	6	4.3
3+	17	4.2
Unknown	21	
**Subtype**		
LUM-A	17	5.05
LUM-B	10	4.5
TNBC	8	4.6
HER2+	13	4.07
Unknown	21	
**Tissue type**		
Normal	12	6.2
DCIS	02	8
Primary tumor	46	4.6
LN metastases	36	3.9
**Grade**		
I	17	4.06
II	48	4.02
III	02	4
Unknown		

RT2 Array

		DOX	DOX	Teniposide	Teniposide	DOX+Teniposide	DOX+Teniposide
Position	Gene	Control Group	Control Group	Test Group 1	Test Group 1	Test Group 2	Test Group 2
A01	AHNAK	24.87063026	25.56221771	25.36950684	25.53912926	25.74189949	25.91060829
A02	AKT1	24.19392967	24.92717934	24.82381439	24.95908356	24.86774063	24.90701103
A03	BMP1	28.12591362	29.02685547	28.42687035	28.63952255	28.4596386	28.6678772
A04	BMP2	31.30188942	32.20625687	32.25372696	32.28382492	32.45786667	32.63058853
A05	BMP7	39.43143463	36.92239761	36.98490524	35.91166306	36.17647552	37.38977051
A06	CALD1	25.37075424	25.92665291	26.25923157	26.2388382	26.38348007	26.42619133
A07	CAMK2N1	26.66463089	27.11363792	27.56232452	27.62007332	27.20334244	27.00463486
A08	CAV2	25.41454887	26.19514847	26.40225792	26.39068794	26.25295067	26.18991852
A09	CDH1	32.78691864	33.27393341	30.87968826	30.95881844	31.59507179	30.9235096
A10	CDH2	33.28096771	33.49031067	32.17519379	31.97125626	32.53765488	32.66656113
A11	COL1A2	35.6661644	36.71207428	36.63647842	35.88016891	37.50819397	34.61646271
A12	COL3A1	33.63405228	34.06332397	32.1666069	31.95306396	32.27146912	31.80145645
B01	COL5A2	32.23841095	32.37587357	30.54700279	30.68490982	31.60148621	30.96518707
B02	CTNNB1	26.54693794	27.41736603	27.81355095	27.73791504	27.83933067	27.74936867
B03	DSC2	29.69991493	30.66831017	29.96657753	29.87551689	30.0413456	29.96734238
B04	DSP	24.93392372	25.59878922	24.93489647	24.98438072	25.18700218	25.08794975
B05	EGFR	22.77663994	23.47132492	25.45104599	25.63413048	25.37086296	25.39418221
B06	ERBB3	27.18776894	27.84478569	27.41981125	27.59189034	27.17396545	27.14151764
B07	ESR1	31.79986954	31.22478867	31.9815464	31.81522751	31.59975052	31.20059776
B08	F11R	25.91975403	26.77187347	27.13326645	27.26077843	26.80159378	26.79380989
B09	FGFBP1	34.58132172	33.98546219	34.45537567	33.68854904	33.90423584	33.66027832
B10	FN1	26.26541901	27.06995392	25.6875248	25.85517311	26.2442627	26.24309921
B11	FOXC2	32.70302963	32.77578354	32.45904922	32.37791443	32.17259598	32.38897705
B12	FZD7	26.96549416	27.96730804	27.70210075	27.81910896	27.59765816	27.68930054
C01	GNG11	27.34964371	27.78325462	26.8170681	26.93666458	27.03829765	26.93942642
C02	GSC	36.00057602	35.89482498	36.35073853	35.3597641	35.61673355	35.43812561
C03	GSK3B	24.79937935	25.42056847	26.7957592	26.78344345	26.78481102	26.63247299
C04	IGFBP4	21.30339622	22.15314293	22.85314178	23.08329582	22.89628983	22.9616375
C05	IL1RN	37.37347031	36.82640457	35.98405457	35.13395691	39.72524643	35.67243958
C06	ILK	22.65719604	23.37059021	23.70672417	23.81349373	23.75526237	23.71356392
C07	ITGA5	24.95021057	25.56607056	25.7428894	25.96077347	25.56297302	25.6360569
C08	ITGAV	26.19270515	26.92244148	26.78841019	26.8191452	26.85176849	26.82509995
C09	ITGB1	20.97551346	21.74646187	22.9814167	22.92029572	22.72114182	22.63923836
C10	JAG1	25.30245972	25.94999886	25.62720299	25.73918343	25.91144943	25.92114258
C11	KRT14	30.70586014	30.34134293	28.98682022	28.91286469	29.1639843	29.0712204
C12	KRT19	21.84496498	22.54026604	23.68671417	23.54497528	23.2383728	23.17289734

D01	KRT7	27.17110825	27.66020203	27.52833748	27.85613251	27.90146828	27.62319565
D02	MAP1B	23.61366081	24.46830559	24.19601059	24.18133545	24.36019325	24.55918694
D03	MMP2	36.22344971	35.80561829	34.99130249	35.9549942	35.51265717	35.74162292
D04	MMP3	29.22077942	30.10331345	28.0012989	27.97643089	28.18925285	28.01217842
D05	MMP9	33.13492966	33.84745407	31.90945435	31.99288559	32.09708023	31.82517242
D06	MSN	21.225914	22.10625839	23.73663902	23.81240845	23.44425774	23.43525887
D07	MST1R	29.43330193	29.98615456	29.84194756	29.76494789	29.60799408	29.35685921
D08	NODAL	33.81418228	34.26325607	32.98072433	33.84522629	34.42729187	34.60913086
D09	NOTCH1	29.87999344	30.67950249	30.48552322	30.5087204	30.60051346	30.4472847
D10	NUDT13	29.43769646	29.58609772	30.20576477	30.12320328	30.01510811	30.04562187
D11	OCLN	25.81695175	26.38870811	26.41709709	26.41464233	26.34105873	26.28255463
D12	PDGFRB	31.74290276	32.82933807	31.42708778	31.40186882	31.62349319	31.51659966
E01	PLEK2	27.00468826	27.14066315	27.52466965	27.83490753	27.4095726	27.22118378
E02	DESI1	25.1111412	25.75836754	25.57644272	25.67430496	25.60498619	25.70321846
E03	PTK2	24.03830147	24.96083832	26.86215782	26.89201927	26.51113892	26.44730759
E04	PTP4A1	23.84806442	24.77909279	24.73665237	24.69176483	24.56957245	24.44826698
E05	RAC1	22.14342117	22.96061325	23.3640976	23.33239365	23.4490509	23.3984375
E06	RGS2	28.63101196	29.375597	28.06376076	27.99843407	28.1722641	28.0021019
E07	SERPINE1	22.72914696	23.46509933	25.36684227	25.53262901	24.97601128	25.02268982
E08	GEMIN2	25.58858109	26.15583611	26.43433571	26.454916	26.23500061	26.21269608
E09	SMAD2	25.49017143	26.2442379	26.98750877	27.0915947	26.96715164	26.94381332
E10	SNAI1	33.97315216	34.56003571	31.92317963	32.03244781	31.89165306	31.93782043
E11	SNAI2	27.24470139	27.72274971	27.51341057	27.52681732	27.78413391	27.84940529
E12	SNAI3	31.52820969	31.90444183	31.22402191	31.23108673	31.01119614	30.92203903
F01	SOX10	35.44900131	35.07096863	35.01335907	34.8669548	35.52598953	35.04347992
F02	SPARC	30.7524662	31.21542931	31.22432137	31.17699814	30.55560493	30.85004807
F03	SPP1	34.84855652	36.29874802	33.08208084	34.5681572	33.68546295	34.87005615
F04	STAT3	23.09323311	24.00046921	23.99683189	24.08447456	23.92785263	23.91691399
F05	STEAP1	25.98139954	26.56078529	27.75190163	27.81797791	27.41533279	27.50044823
F06	TCF3	25.79071808	26.29044533	26.55239868	26.40225792	26.4525795	26.35486412
F07	TCF4	29.89530563	29.24694443	29.94738579	29.20081329	29.09609222	28.96374512
F08	TFPI2	23.98389816	24.48003387	23.51594353	23.50390816	23.01697159	22.953722
F09	TGFB1	23.86709404	24.52031517	24.5362072	24.80808449	24.27475357	24.3926239
F10	TGFB2	24.95836258	25.58105469	26.89778709	26.92900658	27.04703712	26.94502068
F11	TGFB3	30.96478271	31.23422623	30.24747086	30.49806595	30.91172981	30.58006859
F12	TIMP1	25.44390869	25.94264603	25.95964622	26.01759911	25.543993	25.59519196
G01	TMEFF1	27.39425087	28.19210625	28.61825371	28.62262154	28.55065346	28.31926727
G02	TMEM132A	27.23928452	28.03660393	26.79410362	27.03924179	26.61492157	26.78466034
G03	TSPAN13	27.41596413	28.00699043	27.01458931	27.13139725	26.35052681	26.49999046
G04	TWIST1	40	39.32951355	40	37.31115723	37.00740814	37.0691452
G05	VCAN	34.17805481	34.94039917	34.9508934	34.54312134	33.43137741	33.63816071

G06	VIM	17.94638252	18.58776093	20.07986259	20.12722588	19.52247429	19.53245735
G07	VPS13A	26.24272537	26.95441437	27.75121498	27.68013573	27.80965233	27.79987717
G08	WNT11	32.59757996	32.04106903	32.45930099	32.14914322	32.14269257	32.12544632
G09	WNT5A	31.29364204	31.26425171	31.00376701	31.20751762	31.60814095	31.56131554
G10	WNT5B	25.21336174	25.566782	25.84340477	26.03237534	25.77361488	25.82706642
G11	ZEB1	25.87361336	26.68874741	27.90843391	27.88847351	27.66387939	27.54659462
G12	ZEB2	27.85351563	28.9424839	29.69888306	29.54069519	29.2989006	29.38592529
H01	ACTB	17.94501686	18.64984131	19.80618095	19.8306179	19.67798996	19.69781113
H02	B2M	21.6555748	21.96126556	22.01630783	21.9559288	21.88479042	21.86646652
H03	GAPDH	19.64247131	20.15379333	20.98754501	21.07762527	20.90807915	20.93336105

<u>Supplemental Information: Sequence #1</u>

NMI 619 promoter sequence

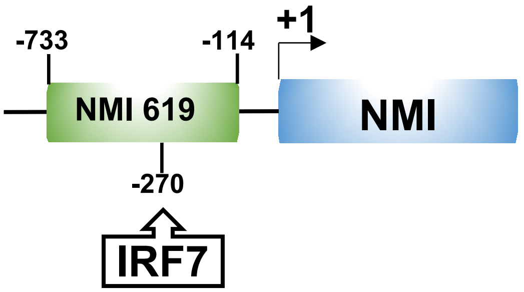

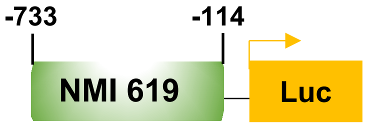

(-733)
GGATTAAGAGACACTGCAGTTCTTGAAATAAACCAGATGTTTGAATTTTCAAAGGAAAAGTTCACACCTA
GTGAAGTATTTATTTACAAGCTCCACAACAAAACAAGAGATTATAAGAAAACTTTCAAGGGACATGGGT
CACAGAAACCAACAGGAACACCACAGCTTTCCCAACTTCATGGCCCCGGGGGATGTTACAAAAGACACA
GAAACGCCGATCTTGTGCTGACTGTAAACATTTGTCAACTAGTGTTACAATGACTAAGAAATTACAAAAG
AGTTACAATCTGATGAAGAGCGATTCCTGCTTTGACTTTATTTGGTTTCTATCCACTTTTCCAACATACCTT
ATAAAATACATGCAAAAAACTGGTTTTAGAAAGCGTTCCCTAAAATTAGTGTGTACGGAAACTTCAGGTA
TACTTCTGCTTTCCTCCGCAAAACCACCCCTTTTTAAA**<u>AATCGAAAGTGAAATTAGTTT</u>**TTTTTTCTGTTA

IRF7

GTGACTAATCATTGGAGACAACCATGTTTAGTATTTGAGCATTGGTTAAATGCTAAAGAAAAATCGCCGT
TAAAGCAGTTTTCTTTTTCACTGTCTTTTTCTTTTCGCGGGGAACCCAGCTGTTCCTG (-114)

Supplemental Information: Sequence #2

Homo sapiens RNA, 45S pre-ribosomal 5 (RNA45SN5), ribosomal RNA
NR_046235

TCCCCAGATGTAGTGAAAGCAGGTAGATTTGCCTTGCCTGGACTTGCCTGGCCTTGCCTTTTCTTTCTTTC
TTTCTTTATTACTTTCTCTTTTTCTTCTTCTTCTTCTTCTTCTTCTTCTTCTTCTTCTTCTTCTTCTTCTTCTT
CTTCTTCTTTTTTTTTTGAGACAGAGTTTCACTCTTGTTGCCCAGGCTAGAGGGCAATGGCGCGATCTCGG
CTCACCGCACCCTCCGCCTCCCAGGTTCAAGCGATTCTCCTGCCTCAGCCTCCTGATTAGCTGGGATTACA
GGCATGGGCCACCGTGCCTGGCTG

ZEB2 binding site

ATGTTTGTACTTTTAGTAGAGACGGTGTTTTTCCATGTTGGTCAGGCTGGTCTCCCACTCCCAACCT**CAGG**
TGGTCCGCCTGCCTTAGCCTCCCAAAGTGCTGGGATGACAGGCGTGAGCCACCGCGCCCAGCCTCTCTC
TCTCTCTCTCTCTCTCTCTCTCTCTCTCTCTCTCGCTCGCTTGCTTGCTTTCGTGCTTTCTTGCTTTCCCGT
TTTCTTGCTTTCTTTCTTTCTTTCGTTTCTTTCATGCTTGCTTTCTTGCTTGCTTGCTTGCTTTCGTGCTTTCTT
GCTTTCCTGTTTTCTTTTTCTTTCTTTCTTTCTTTCTTTCTTTTGTTTCTTTCTTGCTTGCTTTCTTGCTTGCTTG
CTTGCTTTCGTGCTTTCTTGCTTTCCTGTTTTCTTTCTTTCTTTCTTTCTTGCTTGCTTTCTTGCTTGCTTGCTT
TCGTGCTTTCTTGTTTTCTCGCTTTCTTTCTTTCTTTTGTTTCTTTCCTGCTTGCTTTCTTGCTTGATTGCTTTC
GTGCTTTCTTGCTTTCTTGTTTTCTTTCTTTCTTTTGTTTCTTTCTTTCTTGCTTCCTTGTTTTCTTGCTTTCTT
GCTTGCTTGCTTTCGTGCTTTCTTGTTTTCTTGCTTTCTTTCTTTTGTTTCTTTCTTGCTTGCTTTCTTGCTTCC
TTGTTTTCTTGCTTTCTTGCTTGCTTGCTTTCGTGCTTTCTTTCTTGCTTTCTTTTCTTTCTTTCTTTTCTTTTTC
TTTCTTTCTTGCTTTCTTTTCTTTCATTCATTCATTCTTTCTTTCTTTCCTTTCTTTCTTTCTTTCTTTCTATCTTT
CTTTCTTTCTTTCTTTCTTTCTTTCTTTCTTTCTTTCTGTTTCGTCCTTTTGAGACAGAGTTTCACTCTTGTTTC
CACGGCTAGAGTGCAATGGCGCGATCTTGGCTCACCGCACCTTCCGCCTCCCGGGTTCGAGCGCTTCTCC
TGCCTCAGCCTCCCGATTAGCGGGGATTACAGGGAGGCACCCCCACGCCTGGCTTGGCTGATGTTTGTG
TTTTTAGTAGGCACGCCGTGTCTCTCCATGTTGCTCAGGCTGGTCTCCAACTCCCGACCTCCTGTGATGCG
CCCACCTCGGCCTCTCGAAGTGCTGGGATGACGGGCGTGAGCCACCGTGCCCGGCCTGTTGACTCATTT
CGCTTTTTTATTTCTTTCGTTTCCACGCGTTTACTTATATGTATTAATGTAAACGTTTCTGTACGCTTATATG
CAAACAACGACAACGTGTATCTCTGCATTGAATACTCTTGCGTATGGTAAATACGTATCGGTTGTATGGA
AATAGACTTCTGTATGATAGATGTAGGTGTTTGTGTTATACAAATAAATACACATCGCTCTATAAAGAAG
GGATCGTCGATAAAGACGTTTATTTTACGTATGAAAAGCGTCGTATTTATGTGTGTAAATGAACGAGCGT
ACGTAGTTATCTCTGTTTTCTTTCTTCCTCTCCTTCGTGTTTTTCTTCCTTCCTTTCTTCCTTTCTCTCCTTCTT
TAGGTTTTTCTTCCTCTCTTCCTTTCCTTCTTTCTCTCTTTCTGTCCTTTTTTCCTTCGTGCTTTATTTCTCTTTC
GTTCCCTGTGTTTCCTTCTTTTTTCTTTCCTCTCTGTTTCTTTTTCCCTTCTTTCCTTCGTTTCTTTCCTCATTCT
TTCTCTCTTTTTCGTGTTTCTTTCCTTCCCGTCTGTCTTTTAAAAAATGGAGTGTTTCAGAAGTTTACTTTGT
GTATCTACGTTTTCTAAATTGTCTCTCTTTTCTCCATTTCTTCCTCCCTCCCTCCCTCCCTCCCTCCCTGCTC
CCTTCCCTCCCTCCTTCCCTTTCGCCATCTGTCTCTTTTCCCCACTCCCCTCCCCCCGTCTGTCTCTGCGTGG
ATTCCGGAAGAGCCTACGCATTCTGCCTCTCCGTGTGTCTGCAGCGACCCGCGACCGAGTCCTTGTGTGT
TCTTTCTCCCTCCCTCCCTCCCTCCCTCCCTCCCTCCCTCCCTGCTTCCGAGAGGCATCTCCAAACACCCAC
GCGCCGTGGGTTGTCTTCTGACTCTGTCGCGGTCGAGGCAGAGACGCGTTTTGGGCACCGTTTGTGTGG
GGTTGGGGCAGAGGGGCTGCGTTTTCGGCCTCGGGAAGAGCTTCTCGACTCACGGTTTCGCTTTCGCGG
TCCACGGGCCGCCCTGCCAGCCGGATCTGTCTCGCTGACGTCCGCGGCGGTTGTCGGGCTCCATCTGGC
GGCCGCTTTGAGATCGTGCTCTCGGCTTCCGGAGCTGCGGTGGCAGCTGCCGAGGGAGGGGACCGTCC
CCGCTGTGAGCTAGGCAGAGCTCCGGAAAGCCCGCGGTCGTCAGCCCGGCTGGCCCGGTGGCGCCAGA

GCTGTGGCGCGTCGCTTGTGAGTCACAGCTCTGGCGTGCAGGTTTATGTGGGGGAGAGGCTGTCGCTG
CGCTTCTGGGCCCGCGGCGGGCGTGGGGCTGCCCGGGCCGGTCGACCAGCGCGCCGTAGCTCCCGAG
GCCCGAGCCGCGACCCGCGGGGACCCGCCGCGCGTGGCGCGGGAGGCTGGGGACGCCCTTCCCGGCC
CGGTCGCGGGTCCGCGCTCATCCTGGCCGTCTGAGGCGGCGGCCGAATTCGTTTCCGAGTCCCCGTGGG
GAGCCGGGGACCGTCCCGCCCCCGTCCCCCGGGTGCCGGGGAGCGGTCCCCGGGCCGGGCCGCGGTCC
CTCTGCCGCGATCCTTTCTGGCGAGTCCCCGTGCGGAGTCGGAGAGCGCTCCCTGAGCGCGCGTGCGGC
CCGAGAGGTCGCGCCTGGCCGGCCTTCGGTCCCTCGTGTGTCCCGGTCGTAGGAGGGGCCGGCCGAAA
ATGCTTCCGGCTCCCGCTCTGGAGACACGGGCCGGCCCCCTGCGTGTGGCACGGGCGGCCGGGAGGGC
GTCCCCGGCCCGGCGCTGCTCCCGCGTGTGTCCTGGGGTTGACCAGAGGGCCCCGGGCGCTCCGTGTGT
GGCTGCGATGGTGGCGTTTTTGGGGACAGGTGTCCGTGTCGCGCGTCGCCTGGGCCGGCGGCGTGGTC
GGTGACGCGACCTCCCGGCCCCGGGGGAGGTATATCTTTCGCTCCGAGTCGGCATTTTGGGCCGCCGG
GTTATT**GCTGAC**ACGCTGTCCTCTGGCGACCTGTCGCTGGAGAGGTTG

Transcription start

REAGENT or RESOURCE	SOURCE	IDENTIFIER
**Antibodies**		
Anti-IRF-7	Cell Signaling Technologies, Danvers, MA	Cat# 4920S
Anti-ZEB2	Bethyl Labs, Montgomery, TX	Cat# A302-474A
Anti-ZEB2	MilliporeSigma, St. Louis, MO	Cat# HPA003456-100UL
Anti-Fibrillarin	Abcam, Cambridge, UK	Cat# ab166630
Anti-Rabbit Alexa Fluor 594	ThermoFisher, Waltham, MA	Cat# A11012
Anti-Mouse Alexa Fluor 488	ThermoFisher, Waltham, MA	Cat# A11001
Anti-Tubulin HRP	Cell Signaling Technologies, Danvers, MA	Cat# 12351S
Anti-NMI clone 9D8	MilliporeSigma, St. Louis, MO	Cat# WH0009111M1-100UG
Anti-ZEB1	Novus Biologicals, Littleton, CO	Cat# NBP1-05987
Anti-Phospho-Histone H2A.X (Ser139)	Cell Signaling Technologies, Danvers, MA	Cat# 9718s
Anti-p53 ser15	Cell Signaling Technologies, Danvers, MA	Cat# 9284
Anti-p53	Cell Signaling Technologies, Danvers, MA	Cat# #30313S
Anti-NFκB p65	Millipore Sigma, St. Louis MO	Cat# 06-418
Anti- Phospho-NF-κB p65 (Ser536)	Cell Signaling Technologies, Danvers, MA	Cat# 3033
Anti-Laminin-5, clone D4B5, Alexa Fluor 488 conjugated	MilliporeSigma, St. Louis, MO	Cat# MAB19562X
**Plasmids/Lentivirus**		
Firefly luciferase +eGFP Lentifect Purified Lentiviral Particles	Genecopeia, Rockville, MD	Cat#LPP-HLUC-Lv201-100-C

TRIPZ Inducible Lentiviral shRNA NMI clone ID V2THS_41116	Horizon Discovery, Waterbeach, UK	Cat# RHS4696-200698634
pGL4.23 [luc2/minP] Vector	Promega, Madison, WI	Cat# E8411
**Reagents, Chemicals, and Recombinant Proteins**		
RPMI-1640 media	ThermoFisher, Waltham, MA	Cat# 22400105
DMEM/F-12 media	ThermoFisher, Waltham, MA	Cat# 11330057
Medium 199	ThermoFisher, Waltham, MA	Cat# 11825015
Hydrocortisone	MilliporeSigma, St. Louis, MO	Cat# H0888-1G
Retinyl Acetate	MilliporeSigma, St. Louis, MO	Cat# R0635-5MG
SurgiFoam	Ethicon, Somerville, NJ	Cat#1972
Fetal Bovine Serum	ThermoFisher, Waltham, MA	Cat# A5256701
Puromycin, Dihydrochloride	MilliporeSigma, St. Louis, MO	Cat# 540411-25MG
INSULIN FROM BOVINE PANCREAS	MilliporeSigma, St. Louis, MO	Cat# I5500-500MG
Doxycycline, Hyclate	MilliporeSigma, St. Louis, MO	Cat# 324385-1GM
Recombinant Human IFN-gamma Protein	R and D Systems, Minneapolis, MN	Cat3 285-IF-100
Bright-Glo Luciferase Assay System	Promega, Madison, WI	Cat# E2610
Luciferase Assay System	Promega, Madison, WI	Cat# E1500
Cultrex 3-D Culture Matrix Reduced Growth Factor Basement Membrane Extract, Pathclear	R and D Systems, Minneapolis, MN	Cat# 3445-005-01
VECTASHIELD PLUS Antifade Mounting Medium with DAPI	Vector Laboratories, Burlingame, CA	Cat# H-2000-2
Teniposide 10mM (1mL in DMSO)	Selleck Chemicals, Houston, TX	Cat# S1787

BioCoat Matrigel Invasion Chambers	Corning, Glendale AZ	Cat# 354480
Fibronectin, Human	Corning, Glendale AZ	Cat# 356008
TaqMan Fast Advanced Master Mix	ThermoFisher, Waltham, MA	Cat# 4444964
Maxima SYBR Green/ROX qPCR Master Mix	ThermoFisher, Waltham, MA	Cat# K0222
**Critical Commercial Assays**		
RNeasy Mini Kit for RNA extraction	Qiagen, Valencia, CA	Cat# 74106
High-Capacity cDNA Reverse Transcription Kit for cDNA synthesis	ThermoFisher, Waltham, MA	Cat# 4374967
CyQUANT Direct Cell Proliferation Assay	ThermoFisher, Waltham, MA	Cat# C35011
Zombie Green Fixable Viability Kit	Biolegend, San Diego, CA	Cat# 423112
SimpleChIP Plus Enzymatic Chromatin IP Kit (Magnetic Beads)	Cell Signaling Technologies, Danvers, MA	Cat# 9005S
RT Profiler PCR Array Human Epithelial to Mesenchymal Transition (EMT)	Qiagen, Valencia, CA	Cat# PAHS-090ZC
Click-iT RNA Alexa Fluor 488 HCS Assay	ThermoFisher, Waltham, MA	Cat# C10327
EnVision +Dual Link System HRP DAB+	Dako	Agilent, Santa Clara, CA

**Deposited Data**<br>All datasets generated in this manuscript are available upon request.<br>Data deposited in Sequence Read Archive: BioProject accession number-PRJNA853372<br><br>Publicly available deposited data we utilized can be found:<br>TCGA Breast Cancer data: Xenabrowser.net<br>Comparison of matched primary and metastasis 4T1.2 syngeneic mammary tumor model of spontaneous bone metastasis: GSE37975<br>Silencing of Irf7 pathways in breast cancer cells promotes bone metastasis through immune escape mechanisms: GSE37828		
**Experimental Models: Organisms/Strains**		
Nmi fl/fl MMTV-Neu K14-Cre	Dr. Rajeev Samant<br>UAB, Birmingham, AL	doi: 10.1038/s41388-017-0037-7
**Software and Algorithms**		
GraphPad Prism Software	San Diego, CA	https://www.graphpad.com
FLOWJO Software	BD Biosciences, San Jose, CA	https://www.flowjo.com/solutions/flowjo
NIS Elements Advanced Research Software	Nikon, Melville, NY	
Gene Set Enrichment Analysis v4.2.3 Mac App	Broad Institute, UC San Diego	https://www.Gsea-msigdb.org
R Studio v4.1.2	Posit Software, Boston MA	
**Primers**		
Human NMI	ThermoFisher, Waltham, MA	Hs00190768_m1

Human CDH1	ThermoFisher, Waltham, MA	Hs01023894_m1
Human CDH2	ThermoFisher, Waltham, MA	Hs00169953_m1
Human KRT14	ThermoFisher, Waltham, MA	Hs00265033_m1
Human SNAI1	ThermoFisher, Waltham, MA	Hs00195591_m1
Human SNAI2	ThermoFisher, Waltham, MA	Hs00950344_m1
Human VIM	ThermoFisher, Waltham, MA	Hs00185584_m1
Human ZEB1	ThermoFisher, Waltham, MA	Hs01566407_m1
Human ZEB2	ThermoFisher, Waltham, MA	Hs00207691_m1
