# Supplementary material for: Targeting EMT using low-dose Teniposide by downregulating ZEB2-driven activation of RNA polymerase I in breast cancer
Source: Cell Death Dis. 2024 May 8;15(5):322. doi: 10.1038/s41419-024-06694-7 (PMC11079014; doi:10.1038/s41419-024-06694-7)
Supplement: Supplementary file 2 — Full length Blots [file 41419_2024_6694_MOESM2_ESM.pptx]

## Slide 1
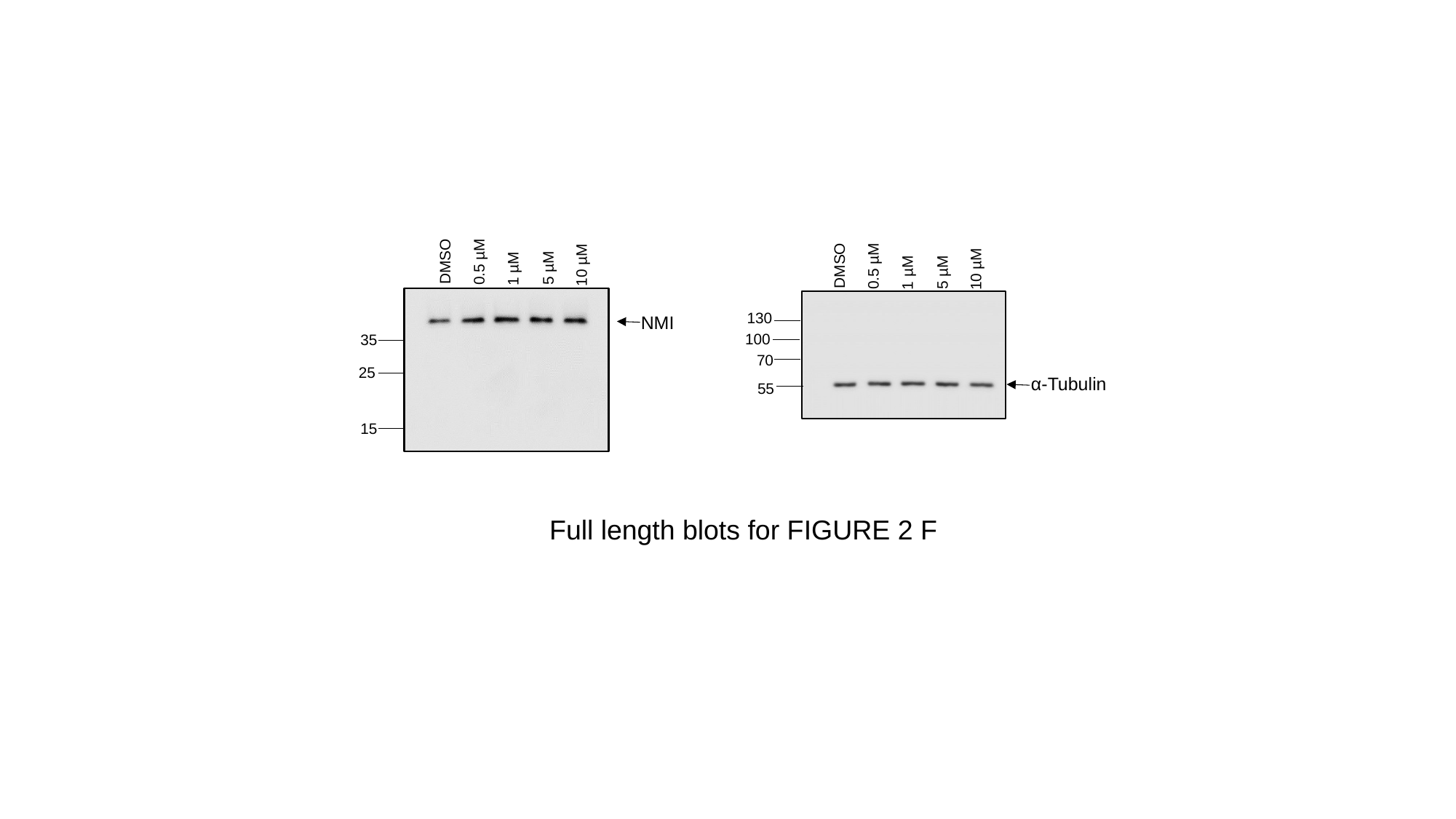

0.5 µM
DMSO
5 µM
1 µM
10 µM
NMI
35
25
15
0.5 µM
DMSO
5 µM
1 µM
10 µM
130
100
70
 α-Tubulin
55
Full length blots for FIGURE 2 F

## Slide 2
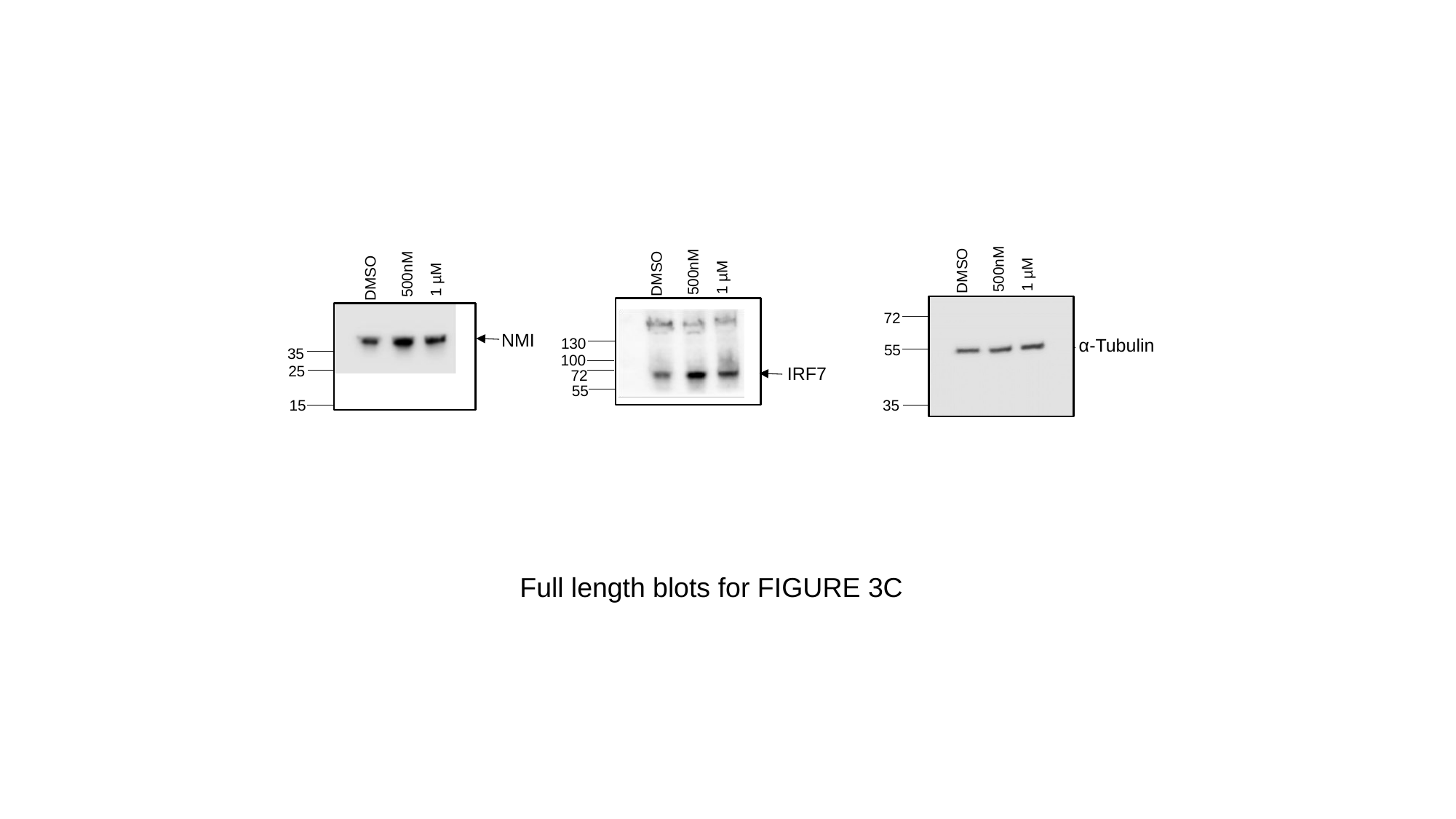

500nM
DMSO
1 µM
72
α-Tubulin
55
35
500nM
DMSO
1 µM
130
100
IRF7
72
55
500nM
DMSO
1 µM
NMI
35
25
15
Full length blots for FIGURE 3C

## Slide 3
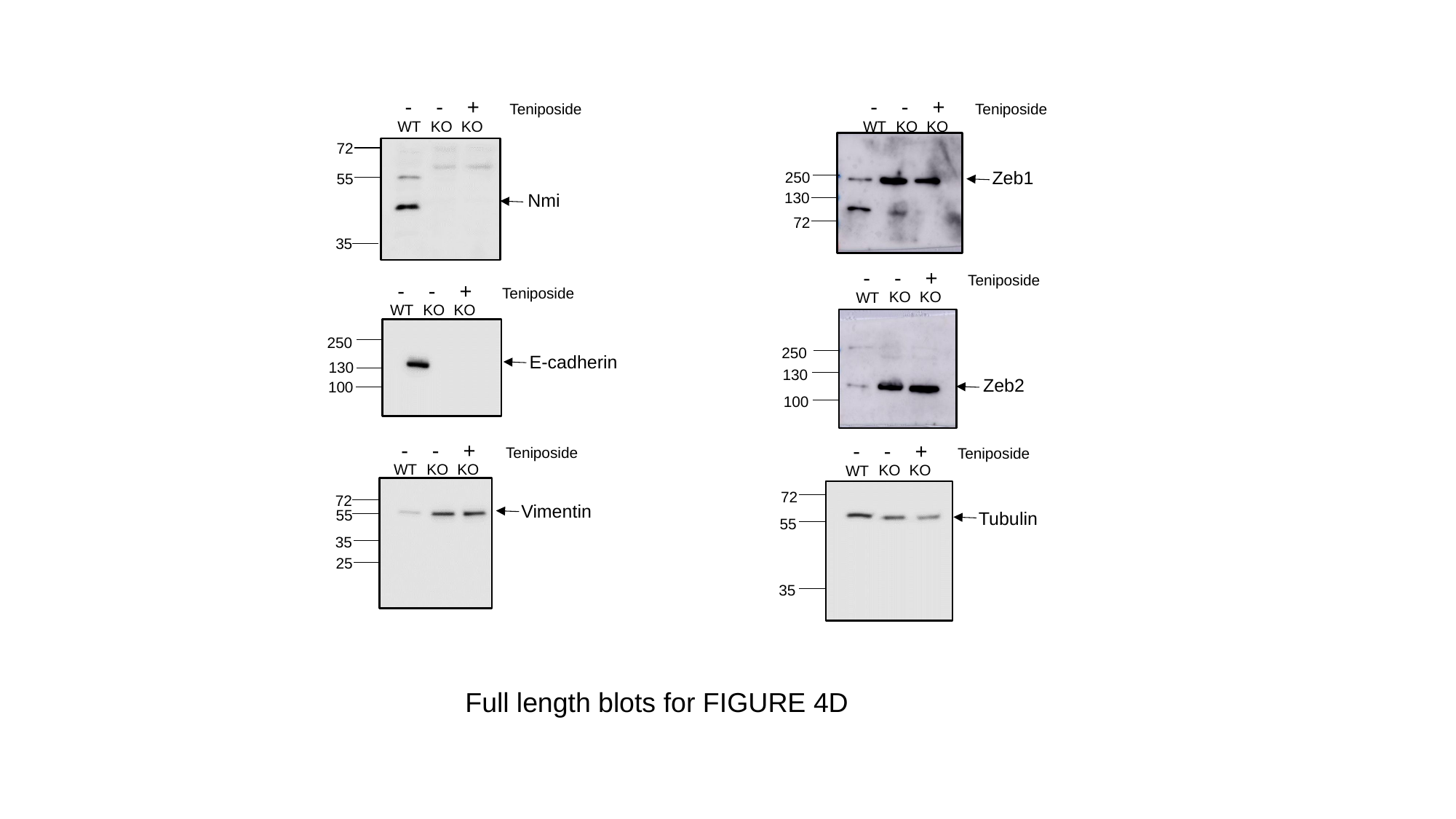

- - + Teniposide
KO
KO
WT
Zeb1
250
130
72
 - - + Teniposide
KO
KO
WT
72
55
Nmi
35
 - - + Teniposide
KO
KO
WT
250
130
Zeb2
100
 - - + Teniposide
KO
KO
WT
250
130
100
E-cadherin
 - - + Teniposide
KO
KO
WT
72
Vimentin
55
35
25
 - - + Teniposide
KO
KO
WT
72
Tubulin
55
35
Full length blots for FIGURE 4D

## Slide 4
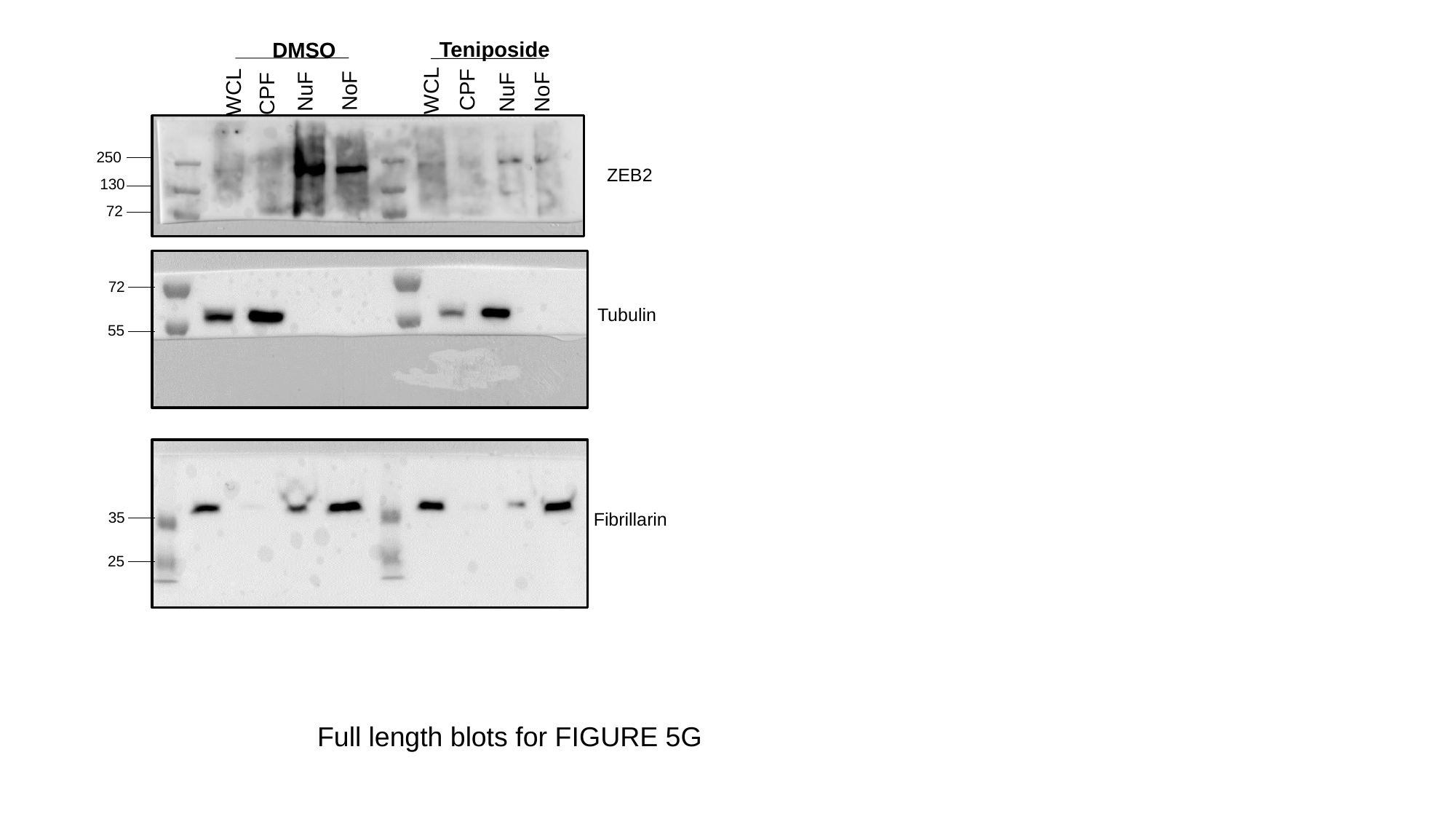

Teniposide
DMSO
CPF
NoF
WCL
NuF
NuF
NoF
WCL
CPF
250
ZEB2
130
72
72
Tubulin
55
35
Fibrillarin
25
Full length blots for FIGURE 5G

## Slide 5
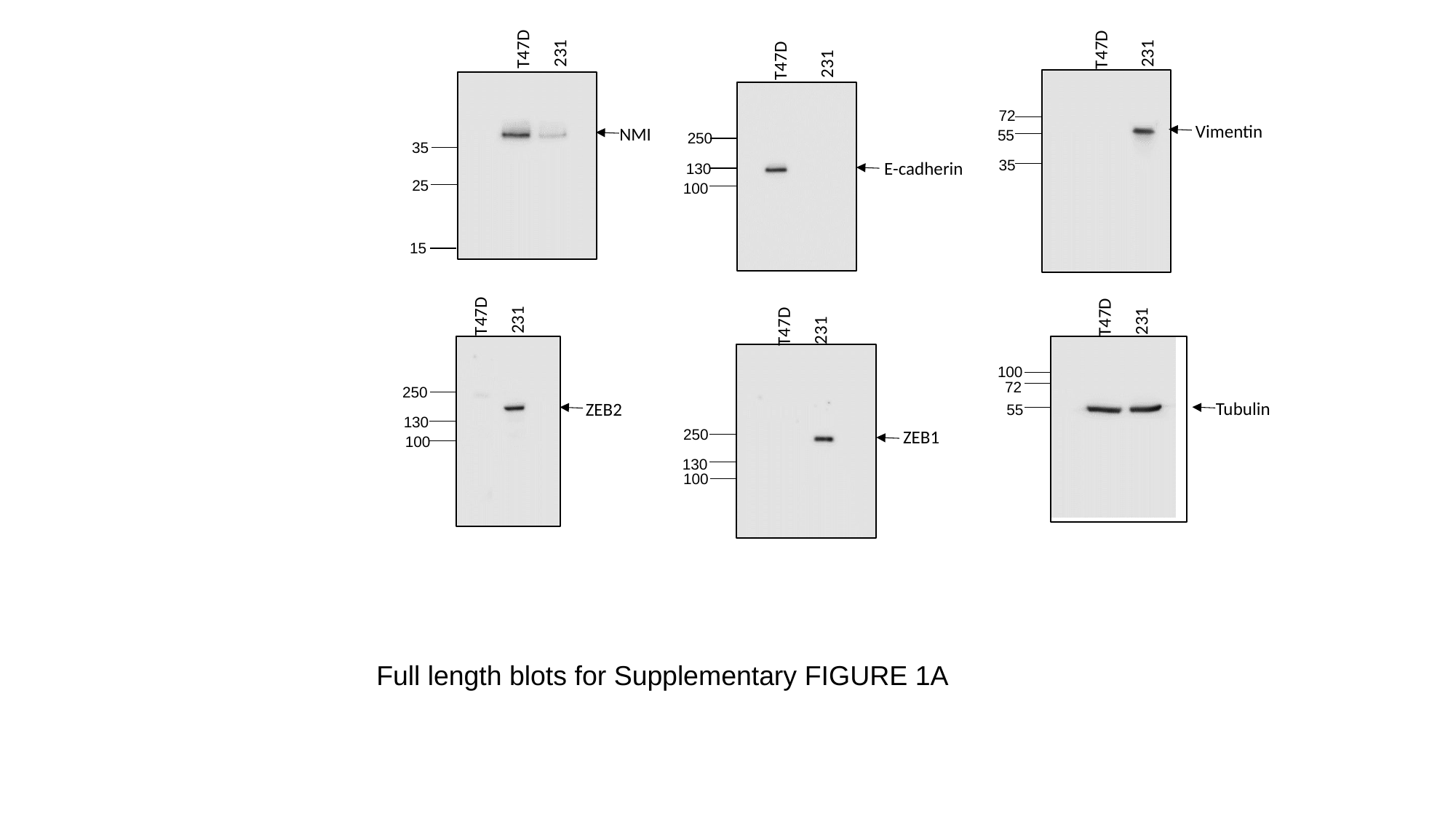

T47D
231
NMI
35
25
15
T47D
231
72
Vimentin
55
35
T47D
231
250
E-cadherin
130
100
T47D
231
250
ZEB2
130
100
T47D
231
100
72
Tubulin
55
T47D
231
250
ZEB1
130
100
Full length blots for Supplementary FIGURE 1A

## Slide 6
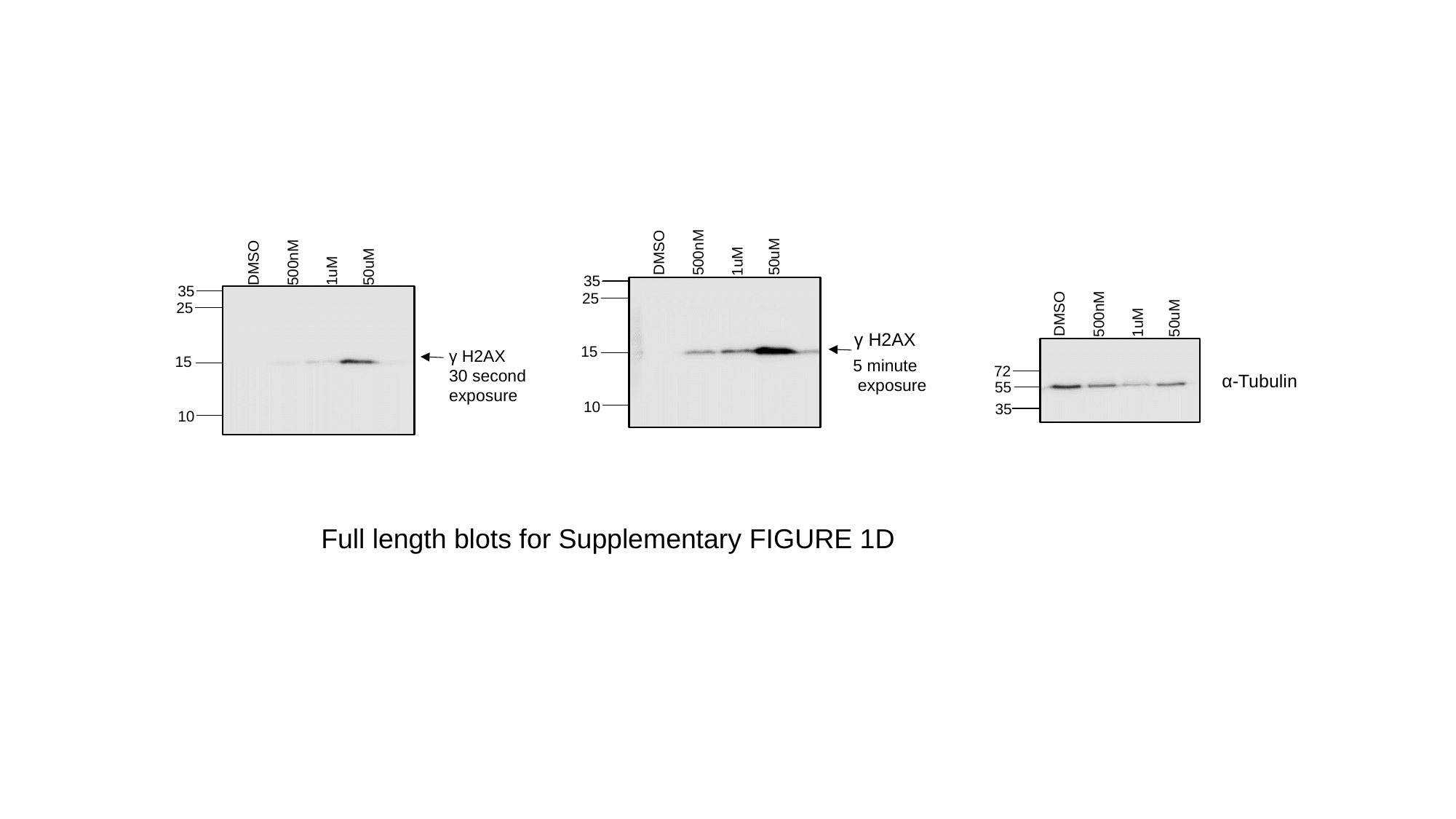

500nM
DMSO
50uM
1uM
35
25
γ H2AX
15
5 minute
 exposure
10
500nM
DMSO
50uM
1uM
35
500nM
DMSO
50uM
1uM
72
 α-Tubulin
55
35
25
γ H2AX
30 second
exposure
15
10
Full length blots for Supplementary FIGURE 1D

## Slide 7
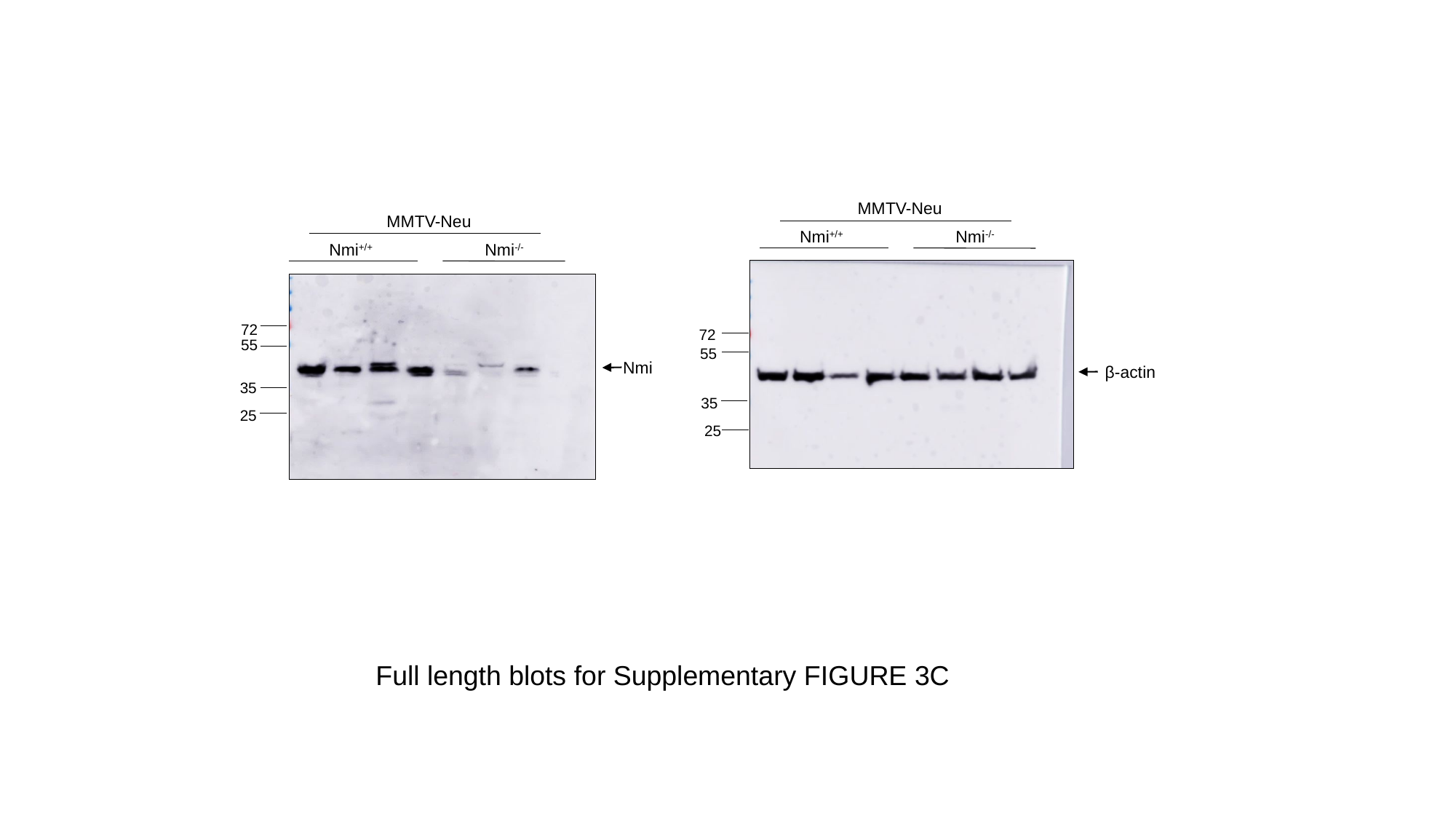

MMTV-Neu
Nmi+/+
Nmi-/-
72
55
β-actin
35
25
MMTV-Neu
Nmi+/+
Nmi-/-
72
55
Nmi
35
25
Full length blots for Supplementary FIGURE 3C

## Slide 8
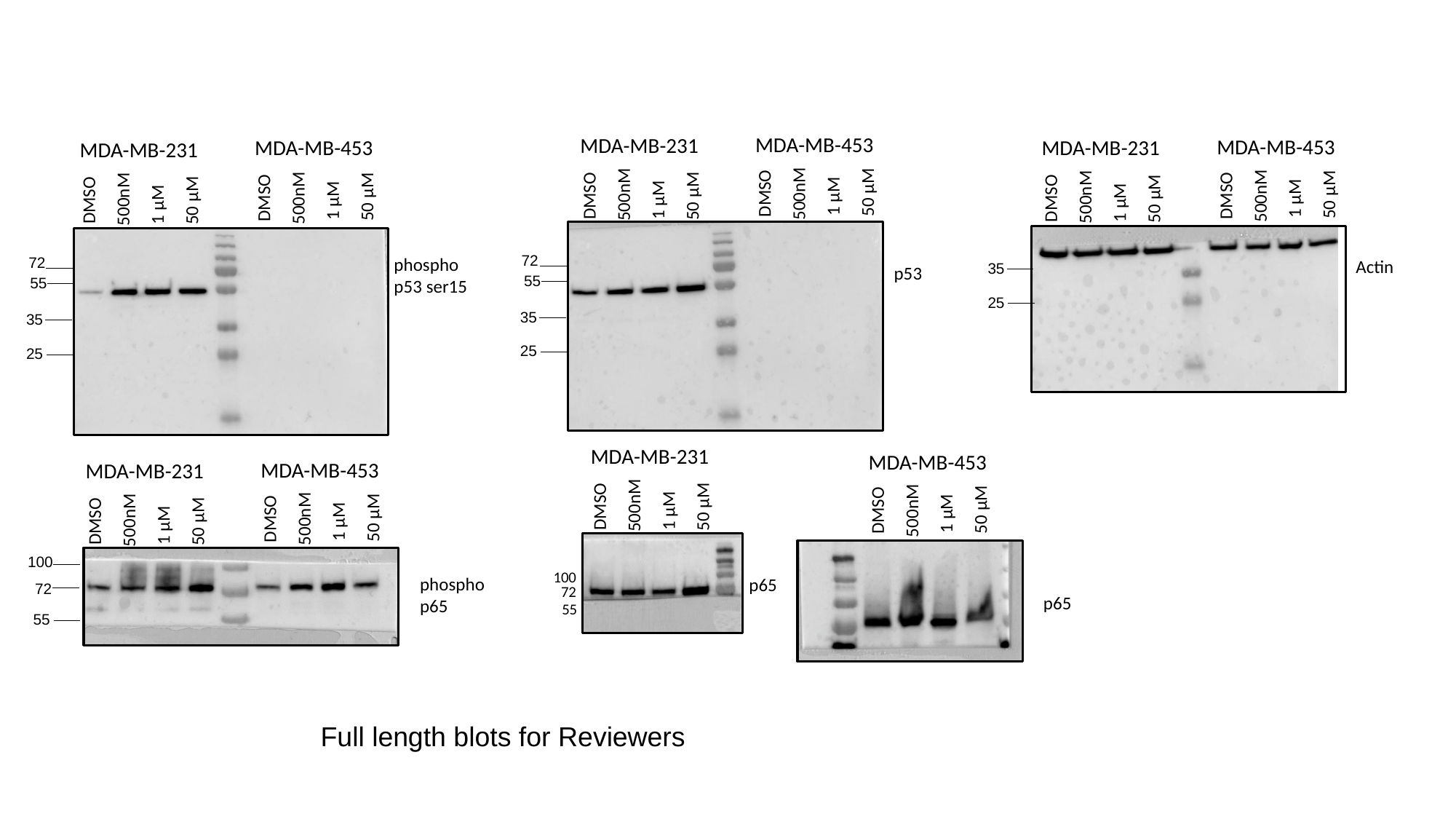

MDA-MB-453
MDA-MB-231
50 µM
500nM
DMSO
500nM
DMSO
50 µM
1 µM
1 µM
MDA-MB-453
MDA-MB-231
50 µM
500nM
DMSO
500nM
DMSO
50 µM
1 µM
1 µM
MDA-MB-453
MDA-MB-231
50 µM
500nM
DMSO
500nM
DMSO
50 µM
1 µM
1 µM
phospho
p53 ser15
72
55
35
25
72
55
35
25
Actin
35
25
p53
MDA-MB-231
MDA-MB-453
500nM
DMSO
50 µM
50 µM
500nM
DMSO
1 µM
1 µM
MDA-MB-453
MDA-MB-231
50 µM
500nM
DMSO
500nM
DMSO
50 µM
1 µM
1 µM
100
100
72
55
phospho
p65
p65
72
p65
55
Full length blots for Reviewers
